# Multimorbidity and adverse outcomes following emergency department attendance: population based cohort study

**DOI:** 10.1136/bmjmed-2023-000731

**Published:** 2024-08-16

**Authors:** Michael C Blayney, Matthew J Reed, John A Masterson, Atul Anand, Matt M Bouamrane, Jacques Fleuriot, Saturnino Luz, Marcus J Lyall, Stewart Mercer, Nicholas L Mills, Susan D Shenkin, Timothy S Walsh, Sarah H Wild, Honghan Wu, Stela McLachlan, Bruce Guthrie, Nazir I Lone

**Affiliations:** 1 Department of Anaesthesia, Critical Care and Pain Medicine, Usher Institute, University of Edinburgh, Edinburgh, UK; 2 Centre for Population Health Sciences, Usher Institute, University of Edinburgh, Edinburgh, UK; 3 Centre for Cardiovascular Science, University of Edinburgh, Edinburgh, UK; 4 Centre for Medical Informatics, Usher Institute, University of Edinburgh, Edinburgh, Edinburgh, UK; 5 Artificial Intelligence and its Applications, University of Edinburgh School of Informatics, Edinburgh, UK; 6 Advanced Care Research Centre, Usher Institute, University of Edinburgh, Edinburgh, Edinburgh, UK; 7 Royal Infirmary of Edinburgh, Edinburgh, Edinburgh, UK; 8 Institute of Health Informatics, University College London, London, UK; 9 The Alan Turing Institute, British Library, London, UK

**Keywords:** Emergency medicine, Epidemiology

## Abstract

**ABSTRACT:**

**Objectives:**

To describe the effect of multimorbidity on adverse patient centred outcomes in people attending emergency department.

**Design:**

Population based cohort study.

**Setting:**

Emergency departments in NHS Lothian in Scotland, from 1 January 2012 to 31 December 2019.

**Participants:**

Adults (≥18 years) attending emergency departments.

**Data sources:**

Linked data from emergency departments, hospital discharges, and cancer registries, and national mortality data.

**Main outcome measures:**

Multimorbidity was defined as at least two conditions from the Elixhauser comorbidity index. Multivariable logistic or linear regression was used to assess associations of multimorbidity with 30 day mortality (primary outcome), hospital admission, reattendance at the emergency department within seven days, and time spent in emergency department (secondary outcomes). Primary analysis was stratified by age (<65 *v* ≥65 years).

**Results:**

451 291 people had 1 273 937 attendances to emergency departments during the study period. 43 504 (9.6%) had multimorbidity, and people with multimorbidity were older (median 73 *v* 43 years), more likely to arrive by emergency ambulance (57.8% *v* 23.7%), and more likely to be triaged as very urgent (23.5% *v* 9.2%) than people who do not have multimorbidity. After adjusting for other prognostic covariates, multimorbidity, compared with no multimorbidity, was associated with higher 30 day mortality (8.2% *v* 1.2%, adjusted odds ratio 1.81 (95% confidence interval (CI) 1.72 to 1.91)), higher rate of hospital admission (60.1% *v* 20.5%, 1.81 (1.76 to 1.86)), higher reattendance to an emergency department within seven days (7.8% *v* 3.5%, 1.41 (1.32 to 1.50)), and longer time spent in the department (adjusted coefficient 0.27 h (95% CI 0.26 to 0.27)). The size of associations between multimorbidity and all outcomes were larger in younger patients: for example, the adjusted odds ratio of 30 day mortality was 3.03 (95% CI 2.68 to 3.42) in people younger than 65 years versus 1.61 (95% CI 1.53 to 1.71) in those 65 years or older.

**Conclusions:**

Almost one in ten patients presenting to emergency department had multimorbidity using Elixhauser index conditions. Multimorbidity was strongly associated with adverse outcomes and these associations were stronger in younger people. The increasing prevalence of multimorbidity in the population is likely to exacerbate strain on emergency departments unless practice and policy evolve to meet the growing demand.

**WHAT IS ALREADY KNOWN ON THIS TOPIC**

**WHAT THIS STUDY ADDS**

- Multimorbidity is prevalent in the emergency department population and people with multimorbidity are more severely ill on presentation and spend longer in the emergency department
- Multimorbidity is strongly associated with adverse outcomes, with a more pronounced relative impact in younger people
- The profile of multimorbidity in younger people reflected a larger burden of mental health and substance misuse conditions than in older people

**HOW THIS STUDY MIGHT AFFECT RESEARCH, PRACTICE, OR POLICY**

- The increasing prevalence of multimorbidity in the population is likely to exacerbate strain on emergency departments unless practice and policy evolve to meet the growing demand
- People with multimorbidity in emergency departments might benefit from improved recognition and tailored care pathways
- Policy change to address drivers of multimorbidity in younger people might be beneficial

## Introduction

 Multimorbidity is when an individual lives with two or more long term conditions.^1^ Prevalence of multimorbidity is increasing over time, in part because of ageing populations, and in part due to better survival from acute conditions.^2^ In studies of general, non-hospitalised populations, multimorbidity has been shown to be associated with poorer health, lower quality of life, and higher rates of healthcare usage.^3^

Multimorbidity is strongly associated with social deprivation and in older people; however, it is still common in younger people,^1^ where its impact may be less well identified. In addition, long term conditions that comprise multimorbidity differ with age,^4^ and might require differing approaches in clinical care. Furthermore, as access to health services and social care services is often determined on age based criteria (often using a threshold of 65 years),^5^ younger people attending emergency departments might experience a greater gap in health and social care services meeting their needs.^6^

While multimorbidity has been well described in population based studies and at a primary care level, minimal literature is available that describes multimorbidity in an emergency department population. Departments are usually busy with limited time to assess and treat people presenting with what are often time critical acute conditions, and so multimorbidity may not be identified. Continuity of care in the emergency department is much lower than in primary care, and clinicians rarely have full access to patients' comprehensive health records. Additionally, emergency department protocols, and subsequent hospital management decisions, tend to focus on single condition protocols and pathways. These factors may present a challenge to holistic and person focused care of those who have multiple long term conditions. In turn, people with multimorbidity might need more time within emergency departments due to their complex needs, and require more resources.

These aspects highlight the importance of carefully describing the epidemiological effects of multimorbidity in the emergency department, and its subsequent associations with patient outcomes, particularly for younger attendees of the emergency department. Our study aimed to evaluate the prevalence of multimorbidity in people attending the emergency department, and its association with mortality, emergency department length of stay, rate of hospital admission, and emergency department reattendance. We also evaluated the prevalence of individual long term conditions, and their associations with mortality; and described the characteristics and outcomes for younger attendees compared with older attendees of the emergency department by undertaking age stratified analyses.

**Figure 1:**
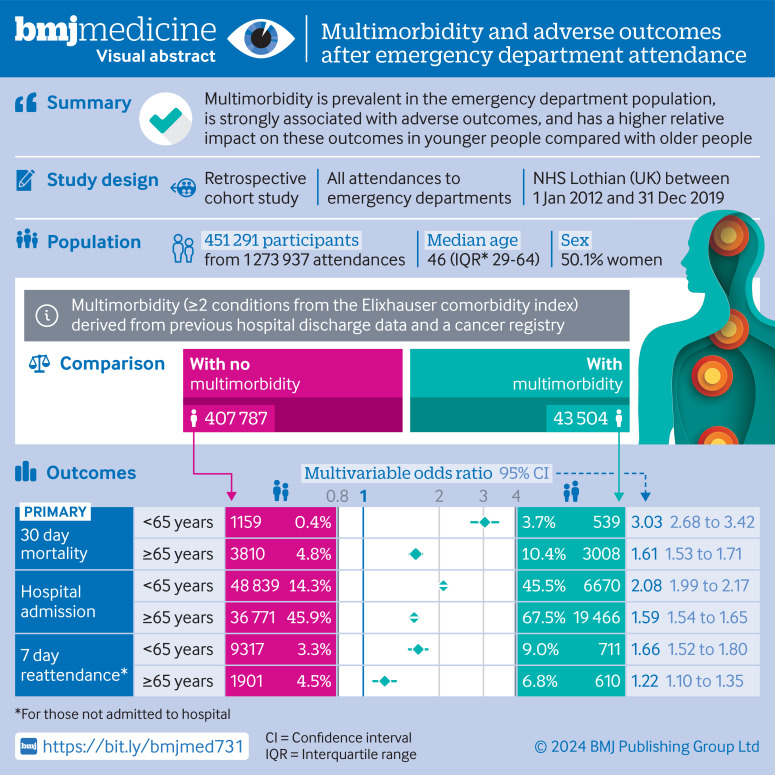
Visual abstract

For the visual abstract of this paper, see figure 1.

## Materials and methods

### Study design and data sources

In this cohort study, data were sourced from Dataloch, which has federated routine healthcare data for all residents of NHS Lothian. Dataloch incorporates a variety of data sources: EDTrak (all emergency department attendances within NHS Lothian, with data entered real-time by clinicians into the electronic health record), national records of Scotland death records (a national database containing all deaths reported in Scotland),^7^ Scottish morbidity records 01 (a national database of all hospital discharges in Scotland),^8^ and Scottish morbidity records 06 (a national registry of all new cancer diagnoses in Scotland).^9^ While no formal quality assessment has been undertaken for fields in the electronic health record, quality assurance levels are high for the national databases because variables in most fields are more than 90% accurate.^10 11^ All data were collected from the electronic health record and national registries. Deidentified data were held in a secure data environment within the NHS (DataLoch, Edinburgh, UK) and was accessed remotely through a dedicated analytical workbench. Individual patient consent was not sought, and only summary data were released from the Secure Data Environment to minimise the risk of disclosure.

Socioeconomic deprivation was defined using the Scottish index of multiple deprivation, a score allocated to all Scottish residents' postcodes, grouped into five groups.^12^ These databases were linked by DataLoch using the community health index, a unique number given to individuals accessing care in Scotland,^13^ with pseudonymised data provided to the researchers for analysis in a safe haven environment.

NHS Lothian is a geographical area in southeast Scotland, covering and providing universal free at point of service healthcare for a population of more than 900 000 people. NHS Lothian has two emergency departments. The distribution of deprivation fifths within NHS Lothian is uneven, with 11% of the population in the most deprived Scottish group and 32% of the population in the least deprived Scottish group in 2019.^14^ A key standard for performance in Scottish emergency departments is that no patient should spend longer than four hours between arriving at the accident and emergency unit and admission, discharge, or transfer.”^15^

### Cohort derivation

We included all adults (≥18 years) presenting to an emergency department within NHS Lothian between 1 January 2012 and 31 December 2019. In cases where multiple presentations for the same individual occurred during the eight year period, a single presentation was selected at random to ensure representative coverage of multimorbidity over the cohort period and to ensure independence of observations in statistical modelling of outcomes.

### Variables

#### Covariates

The primary covariate of interest for this study was multimorbidity, defined as two or more long term conditions, derived using the Elixhauser list of conditions^16^ (an extension of the Charlson comorbidity index^17^ that includes some mental health conditions). This list was modified to merge multilevel long term conditions, such as hypertension with no complications and hypertension with complications into single categories (online supplemental eTable 1). Long term conditions were derived from the International Classification of Diseases, 10th Revision, diagnoses codes using hospital discharge records and the cancer registry using a look back period of five years prior to the index emergency department attendance. In addition to the multimorbidity variable, we evaluated the association between individual long term conditions and outcomes.

Other variables included demographics (age at emergency department attendance, sex, Scottish Index of Multiple Deprivation^12^ categorised into fifths, and ethnic group); previous health status, measured by number of emergency department attendances and hospital admission in the previous year; severity of illness at time of attendance measured by triage category; referral category and mode of arrival; arrival time; month and year of attendance to allow for seasonality; and secular trends^18 19^ (online supplemental eTable 3).

#### Processes and outcomes

The primary outcome was death within 30 days of attending the emergency department. Secondary outcomes included time spent in the emergency department, admission to hospital from the emergency department, and reattendance at the emergency department within seven days. Reattendance was restricted to the cohort of patients discharged from the emergency department alive and not admitted to hospital because patients who died or were admitted to hospital were not at risk or at substantially lower risk of emergency department reattendance within seven days. Other descriptive emergency department processes and outcomes included: time between presentation and healthcare provider initial assessment, proportion leaving emergency department within four hours, discharge destination, level of care patients were were admitted to, hospital length of stay, in-hospital mortality, 24 h mortality, and seven day mortality (online supplemental eTable 2).

### Statistical analysis

The significance level used was 5% with 95% confidence intervals (CI) and two sided P values reported. Data were reported using percentages for categorical variables and median and interquartile range for continuous variables. Baseline characteristics and outcomes were stratified by multimorbidity status, and differences in outcomes by multimorbidity were compared using χ^2^ and Mann-Whitney tests.

Multivariable logistic regression was used to examine the association between multimorbidity and 30 day mortality (primary outcome), accounting for age (as a continuous variable, after assessing for linear association with outcomes), sex, Scottish index of multiple deprivation quintile, ethnic group, triage category, number of previous emergency department attendances, time of day, and month and year of presentation. The primary purpose of the multivariable model was to understand the prognostic effect of multimorbidity, accounting for other prognostic factors, not to draw causal inference. Missing data for ethnic group and Scottish index of multiple deprivation were entered into the model as their own categories. Other variables only had small numbers of missing data; these observations were excluded from the model. Another model using multiple imputation of all missing data using chained equations was performed as a sensitivity analysis. Ten imputed datasets were used and estimates pooled using Rubin's rules.^20^

The prevalence of individual long term conditions and multimorbidity was reported for the whole cohort and stratified by age groups (<65 years and ≥65 years, as per previous multimorbidity literature).^21^ We decided, a priori, to dichotomise age at this threshold for stratified analyses despite, from a statistical perspective, information being lost by dichotomising continuous variables.^22^ This decision was made because patients younger than 65 years generally do not have access to the same range of multidisciplinary services in both healthcare and social care settings. However, we also visualised the age-multimorbidity interaction for the primary outcome entering age as a continuous variable to provide additional insight.

The cohort was then stratified into patients younger than 65 years and those 65 years and older, and modelling repeated, to explore whether the prognostic effect of multimorbidity varied by age (effect modification). Age was entered as an interaction term with multimorbidity, and the P value for this interaction was reported. Kaplan-Meier plots were also produced to assess unadjusted survival following emergency department attendance, stratified by multimorbidity and age category.

The primary covariate of interest, multimorbidity, was also entered into models as individual long term conditions, adjusting for the same covariates, to assess their individual associations with 30 day mortality; multimorbidity was also stratified by age category.

The main multivariable logistic regression models were repeated for the secondary outcomes of hospital admission and seven day reattendance, controlling for the same set of covariates. For length of stay in an emergency department, multivariable linear regression was used. Effect modification of the multimorbidity age association was assessed using the binary age term for each secondary outcome. Additionally, cumulative incidence of emergency department reattendance was plotted, adjusting for the competing risk of mortality, for people who were discharged from emergency department alive and not admitted to hospital. This plot was stratified by multimorbidity, and a combination of multimorbidity and age category.

Data were analysed using R version 4.1.3, including packages tidyverse, finalfit, survival, cmprsk, and mice.^23^

### Patient and public involvement

We worked with a patient and public involvement group based in Edinburgh with long term conditions at different stages of the project. The group provided insights at the stages of formulating the research question and contributed to study design, including outcome selection. The research findings will be disseminated via social media and professional societies.

## Results

**Figure 2:**
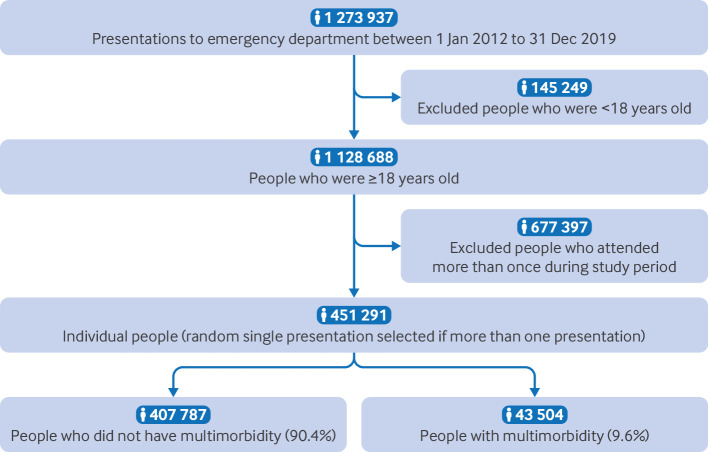
Study's cohort selection

Between 1 January 2012 and 31 December 2019, 1 273 937 attendances were made to an emergency department in NHS Lothian (southeast Scotland). Of these, 451 291 people who were at least 18 years old at date of presentation attended the department 1 128 688 times. In this group, the prevalence of multimorbidity was 9.6% (n=43 504; Figure 2).

The median age of the cohort was 46 years (interquartile range 29-64) and 225 893 (50.1%) were women (table 1). The most common triage category was urgent (n=165 130 (36.6%)). People with multimorbidity versus people who did not have multimorbidity were older (median age 73 years (interquartile range 58-82) *v* 43 (28-60)), more likely to be white (38 464 (88.4%) *v* 313 034 (76.8%)), and more likely to live in the most deprived fifth of postcodes (7214 (16.6%) *v* 56 160 (13.8%)). These people were also more likely to have had two or more emergency department presentations in the previous year (8958 (20.6%) *v* 14 579 (3.6%)), more likely to arrive by emergency ambulance (25 159 (57.8%) *v* 96 693 (23.7%)), and more likely to be triaged into immediate resuscitation (4873 (11.2%) *v* 14 906 (3.7%)) or into very urgent (10 233 (23.5%) *v* 37 627 (9.2%)).

**Table 1:** Baseline characteristics of patients who presented to the emergency department, stratified by multimorbidity status. Data are number (percentage), unless otherwise specified

No. of patients	All (n=451 291)	No multimorbidity (n=407 787)	Multimorbidity (43 504)
**Patient characteristics**			
Age, median (interquartile range) years	46 (29-64)	43 (28-60)	73 (58-82)
Sex:			
Women	225 893 (50.1)	203 776 (50.0)	22 117 (50.8)
Men	225 384 (49.9)	203 997 (50.0)	21 387 (49.2)
Ethnic group:			
African/black	2890 (0.6)	2780 (0.7)	110 (0.3)
Asian	8739 (1.9)	8261 (2.0)	478 (1.1)
Other or multiple groups	14 518 (3.2)	14 154 (3.5)	364 (0.8)
Refused, unknown, or missing	73 646 (16.3)	69 558 (17.1)	4088 (9.4)
White	351 498 (77.9)	313 034 (76.8)	38 464 (88.4)
Scottish index of multiple deprivation:			
1 (most deprived)	63 374 (14.0)	56 160 (13.8)	7214 (16.6)
2	101 736 (22.5)	90 340 (22.2)	11 396 (26.2)
3	80 942 (17.9)	73 266 (18.0)	7676 (17.6)
4	82 596 (18.3)	75 617 (18.5)	6979 (16.0)
5 (least deprived)	114 134 (25.3)	104 223 (25.6)	9911 (22.8)
Scottish index of multiple deprivation unknown	8509 (1.9)	8181 (2.0)	328 (0.8)
**Previous health status**			
Emergency department attendances:			
None in previous year	371 391 (82.3)	347 265 (85.2)	24 126 (55.5)
One in previous year	56 363 (12.5)	45 943 (11.3)	10 420 (24.0)
Two or more in previous year	23 537 (5.2)	14 579 (3.6)	8958 (20.6)
Hospital admissions:			
None in previous year	376 573 (83.4)	361 529 (88.7)	15 044 (34.6)
One in previous year	47 007 (10.4)	34 429 (8.4)	12 578 (28.9)
Two or more in previous year	27 711 (6.1)	11 829 (2.9)	15 882 (36.5)
**Illness severity**			
Referral:			
999 emergency	102 090 (22.6)	81 964 (20.1)	20 126 (46.3)
Flow centre	11 349 (2.5)	9067 (2.2)	2282 (5.2)
GP	27 568 (6.1)	22 826 (5.6)	4742 (10.9)
NHS24	32 710 (7.2)	29 930 (7.3)	2780 (6.4)
Other	18 412 (4.1)	16 631 (4.1)	1781 (4.1)
Self-referral	248 619 (55.1)	238 207 (58.4)	10 412 (23.9)
Unscheduled care services	10 332 (2.3)	8965 (2.2)	1367 (3.1)
Arrival mode:			
Emergency ambulance	121 852 (27.0)	96 693 (23.7)	25 159 (57.8)
Urgent ambulance	8079 (1.8)	5274 (1.3)	2805 (6.4)
Private transport	272 967 (60.5)	260 279 (63.8)	12 688 (29.2)
Public transport	34 666 (7.7)	33 250 (8.2)	1416 (3.3)
Other	13 194 (2.9)	11 800 (2.9)	1394 (3.2)
Triage:			
Immediate resuscitation	19 779 (4.4)	14 906 (3.7)	4873 (11.2)
Very urgent	47 860 (10.6)	37 627 (9.2)	10 233 (23.5)
Urgent	165 130 (36.6)	145 743 (35.7)	19 387 (44.6)
Standard	128 132 (28.4)	122 658 (30.1)	5474 (12.6)
Non-urgent	1188 (0.3)	1148 (0.3)	40 (0.1)
See and treat	67 157 (14.9)	65 788 (16.1)	1369 (3.1)
Medical expected	8522 (1.9)	6906 (1.7)	1616 (3.7)
Suitable for redirection	3227 (0.7)	3143 (0.8)	84 (0.2)
**Time variables**			
Time of attendance:			
0600-1159	114 364 (25.3)	104 279 (25.6)	10 085 (23.2)
1200-1759	162 154 (35.9)	146 237 (35.9)	15 917 (36.6)
1800-2359	124 318 (27.5)	112 345 (27.5)	11 973 (27.5)
0000-0559	50 451 (11.2)	44 922 (11.0)	5529 (12.7)
Month of attendance:			
January to March	108 783 (24.1)	97 784 (24.0)	10 999 (25.3)
April to June	112 486 (24.9)	101 722 (24.9)	10 764 (24.7)
July to September	114 987 (25.5)	104 410 (25.6)	10 577 (24.3)
October to December	115 035 (25.5)	103 871 (25.5)	11 164 (25.7)
Year of attendance:			
2012	53 176 (11.8)	46 539 (11.4)	6637 (15.3)
2013	52 474 (11.6)	46 940 (11.5)	5534 (12.7)
2014	55 238 (12.2)	49 698 (12.2)	5540 (12.7)
2015	53 450 (11.8)	48 290 (11.8)	5160 (11.9)
2016	55 546 (12.3)	50 412 (12.4)	5134 (11.8)
2017	56 759 (12.6)	51 780 (12.7)	4979 (11.4)
2018	59 188 (13.1)	54 106 (13.3)	5082 (11.7)
2019	65 460 (14.5)	60 022 (14.7)	5438 (12.5)

Multimorbidity defined as the presence of two or more long term conditions, derived from the Elixhauser index conditions.

**Figure 3:**
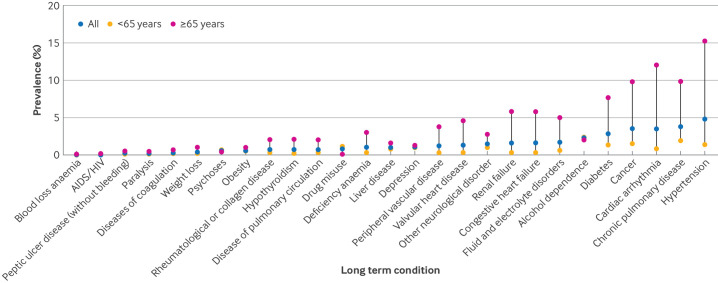
Prevalence of long term conditions stratified by age category (<65 years *v* ≥65 years). Prevalence of Elixhauser conditions, arranged in order of lowest and highest in the whole population (blue).

The number of long term conditions increased with age and social deprivation, but was similar in men and women (online supplemental eFigure 1). Hypertension was the most common long term condition (n=21 496 (4.8%)), followed by chronic pulmonary disease,cardiac arrhythmia, and cancer (figure 3; online supplemental eTable 4).

Three quarters of the cohort were younger than 65 years (n=342 289 (75.8%)), but the prevalence of multimorbidity was greater in patients 65 years and older (28 836 (26.4%) ≥65 years *v* 14 668 (4.3%) <65 years). Most long term conditions were more prevalent in the older population. However, long term conditions of psychiatric and substance misuse were larger in the younger population: alcohol dependence (8371 (2.4%) <65 years *v* 2197 (2.0%) ≥65 years), drug misuse (3691 (1.1%) *v* 57 (0.1%)), psychoses (1966 (0.6%) *v* 464 (0.4%)), and AIDS/HIV (182 (0.1%) *v* 11 (<0.01%); Figure 3, online supplemental eTable 4).

### Processes and outcomes

**Figure 4:**
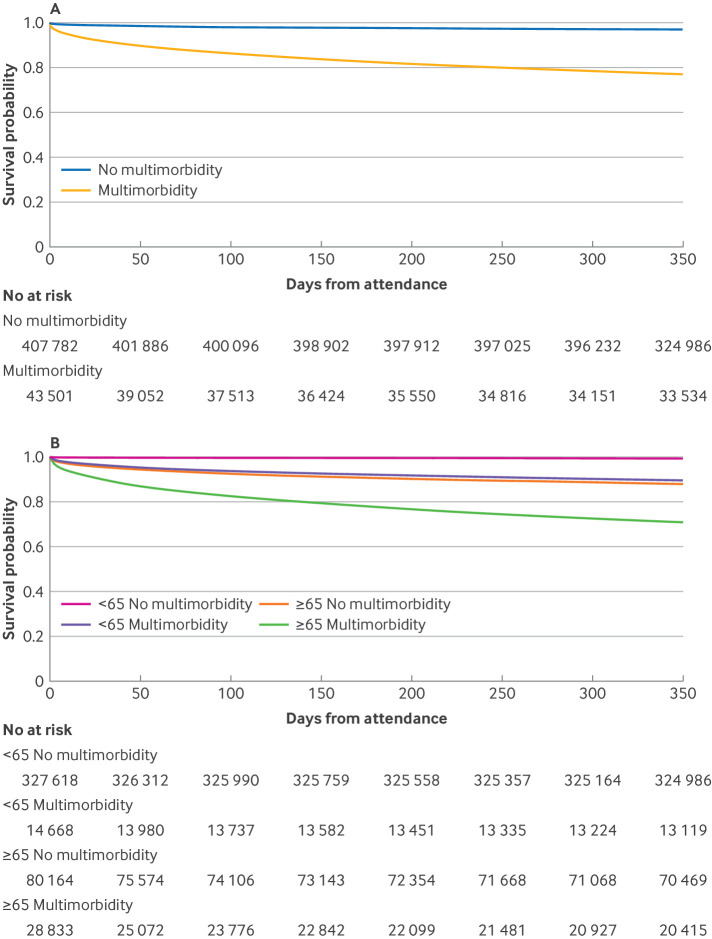
Kaplan Meier plots, stratified by multimorbidity and age Kaplan Meier plots showing survival probability following emergency department attendance, stratified by multimorbidity (**A**) and a combination of multimorbidity and age category (**B**). Graphs are curtailed at 365 days

In comparison to people who do not have multimorbidity, people with multimorbidity had higher mortality within 24 h (743 (1.7%) *v* 1445 (0.4%)), seven days (1901 (4.4%) *v* 3011 (0.7%)), and 30 days of presentation (3547 (8.2%) *v* 4969 (1.2%)) (table 2, table 3, and figure 4A).

**Table 2:** Processes and outcomes in patients presenting to the emergency department, stratified by multimorbidity status

No. of patients	All (n=451 291)	No multimorbidity (n=407 787)	Multimorbidity (n=43 504)	P value
**Departmental outcomes**				
Time (mins) to be seen:				
Median (interquartile range)	76 (40-121)	76.3 (41-122)	68 (34-113)	<0.001
Mean (SD)	84.6 (55.4)	85.4 (55.4)	78.1 (54.6)	—
Seen by HCP within four hours, No. (%)*:				
No	2680 (0.7)	2448 (0.8)	232 (0.6)	0.005
Yes	355 490 (99.3)	318 899 (99.2)	36 591 (99.4)	—
Hours spent in emergency department:				
Median (interquartile range)	3.4 (2.3-4.3)	3.3 (2.3-4.2)	4 (3.2-4.9)	<0.001
Mean (SD)	3.4 (1.6)	3.3 (1.6)	4.3 (1.9)	—
Left emergency department within four hours, No. (%)*:				
No	119 183 (33.3)	100 566 (31.3)	18 617 (50.6)	<0.001
Yes	238 953 (66.7)	220 753 (68.7)	18 200 (49.4)	—
**Discharge outcomes**				
Discharge destination, No. (%):				
Admission to critical care or critical care unit	6231 (1.4)	5049 (1.2)	1182 (2.7)	<0.001
Admission to hospital ward	103 519 (22.9)	78 565 (19.3)	24 954 (57.4)	—
Died before hospital admission	1350 (0.3)	962 (0.2)	388 (0.9)	—
Home	295 950 (65.6)	280 714 (68.8)	15 236 (35.0)	—
Outpatient clinic/ambulatory care	44 241 (9.8)	42 497 (10.4)	1744 (4.0)	—
Level of care admitted to, No. (%):				
Critical care unit	1930 (0.4)	1667 (0.4)	263 (0.6)	<0.001
High dependency units	1756 (0.4)	1285 (0.3)	471 (1.1)	—
Intensive care units	2545 (0.6)	2097 (0.5)	448 (1.0)	—
Non-critical care	103 519 (22.9)	78 565 (19.3)	24 954 (57.4)	—
Not admitted	341 541 (75.7)	324 173 (79.5)	17 368 (39.9)	—
Hospital length of stay (days)†				
Median (interquartile range)	3 (1-8)	2 (1-6.5)	4.5 (1.5-13)	<0.001
Mean (SD)	9.9 (24.8)	8.7 (23.6)	13.7 (28.2)	——
Repeat emergency department presentation within seven days, No. (%)‡:				
No	325 326 (96.3)	309 773 (96.5)	15 553 (92.2)	<0.001
Yes	12 539 (3.7)	11 218 (3.5)	1321 (7.8)	—
**Mortality outcomes**				
Died within 24 h of presentation, No. (%):				
Died	2188 (0.5)	1445 (0.4)	743 (1.7)	<0.001
Survived	449 103 (99.5)	406 342 (99.6)	42 761 (98.3)	—
Died within seven days of presentation, No. (%):				
Died	4912 (1.1)	3011 (0.7)	1901 (4.4)	<0.001
Survived	446 379 (98.9)	404 776 (99.3)	41 603 (95.6)	—
Died within 30 days of presentation, No. (%):				
Died	8516 (1.9)	4969 (1.2)	3547 (8.2)	<0.001
Survived	442 775 (98.1)	402 818 (98.8)	39 957 (91.8)	—
In-hospital mortality†, No. (%):				
Died	6444 (6.1)	3638 (4.6)	2806 (11.0)	<0.001
Survived	98 720 (93.9)	75 993 (95.4)	22 727 (89.0)	—

P value indicates unadjusted significance testing between patients with multimorbidity and patients who did not have multimorbidity. For categorical variables, we used χ2, and for continuous variables, we used Mann-Whitney testing.

* This statistic only applies to patients for whom the four hour rule is applicable.

† This statistic only applies to patients who were admitted to hospital.

‡ This statistic only applies to patients who were discharged alive from emergency department and were not admitted to hospital.

HCP, healthcare professional; SD, standard deviation.

**Table 3:** Logistic regression model for 30 day mortality. Data are No. (%) or odds ratio (95% confidence interval)

Dependent: 30 day mortality	Survived	Died	Odds ratio (univariable)	Odds ratio (multivariable)	Odds ratio (multiple imputation)
Multimorbidity:					
No multimorbidity	402 804 (98.8)	4969 (1.2)	—	—	—
Multimorbidity	39 957 (91.8)	3547 (8.2)	7.20 (6.88 to 7.52, P<0.001)	1.81 (1.72 to 1.91, P<0.001)	1.78 (1.69 to 1.87, P<0.001)
Age, mean (SD)	47.2 (20.6)	75.0 (14.3)	1.07 (1.07 to 1.08, P<0.001)	1.06 (1.06 to 1.06, P<0.001)	1.06 (1.06 to 1.06, P<0.001)
Sex					
Female	221 780 (98.2)	4113 (1.8)	—	—	—
Male	220 981 (98.0)	4403 (2.0)	1.07 (1.03 to 1.12, P=0.001)	1.23 (1.17 to 1.29, P<0.001)	1.23 (1.18 to 1.29, P<0.001)
Ethnic group					
White	344 662 (98.1)	6825 (1.9)	—	—	—
Asian	8686 (99.4)	53 (0.6)	0.31 (0.23 to 0.40, P<0.001)	0.74 (0.55 to 0.97, P=0.037)	0.72 (0.52 to 1.01, P=0.055)
African/black	2880 (99.7)	10 (0.3)	0.18 (0.09 to 0.31, P<0.001)	0.63 (0.31 to 1.13, P=0.158)	0.46 (0.24 to 0.87, P=0.017)
Other/multiple groups	14 478 (99.7)	40 (0.3)	0.14 (0.10 to 0.19, P<0.001)	0.50 (0.35 to 0.67, P<0.001)	0.51 (0.28 to 0.93, P=0.029)
Refused/unknown/missing	72 055 (97.8)	1588 (2.2)	1.11 (1.05 to 1.18, P<0.001)	1.37 (1.29 to 1.46, P<0.001)	—
Scottish index of multiple deprivation:					
5 (least deprived)	112 013 (98.1)	2120 (1.9)	—	—	—
4	81 184 (98.3)	1409 (1.7)	0.92 (0.86 to 0.98, P=0.012)	1.17 (1.09 to 1.26, P<0.001)	1.17 (1.09 to 1.26, P<0.001)
3	79 376 (98.1)	1565 (1.9)	1.04 (0.98 to 1.11, P=0.224)	1.35 (1.25 to 1.44, P<0.001)	1.34 (1.25 to 1.44, P<0.001)
2	99 599 (97.9)	2132 (2.1)	1.13 (1.06 to 1.20, P<0.001)	1.39 (1.30 to 1.48, P<0.001)	1.37 (1.29 to 1.47, P<0.001)
1 (most deprived)	62 131 (98.0)	1239 (2.0)	1.05 (0.98 to 1.13, P=0.148)	1.50 (1.39 to1.62, P<0.001)	1.49 (1.38 to 1.61, P<0.001)
Scottish index of multiple deprivation unknown	8458 (99.4)	51 (0.6)	0.32 (0.24 to0.42, P<0.001)	0.83 (0.62 to 1.10, P=0.211)	—
Triage category:					
Medical expected	8257 (96.9)	265 (3.1)	—	—	—
See and treat	67 148 (100.0)	9 (0.0)	0.00 (0.00 to 0.01, P<0.001)	0.01 (0.01 to 0.02, P<0.001)	0.01 (0.01 to 0.02, P<0.001)
Standard or non-urgent	129 157 (99.9)	163 (0.1)	0.04 (0.03 to 0.05, P<0.001)	0.08 (0.06 to 0.09, P<0.001)	0.08 (0.06 to 0.09, P<0.001)
Urgent	163 039 (98.7)	2091 (1.3)	0.40 (0.35 to 0.46, P<0.001)	0.49 (0.43 to 0.56, P<0.001)	0.48 (0.42 to 0.55, P<0.001)
Very urgent or immediate	61 699 (91.2)	5940 (8.8)	3.00 (2.65 to 3.41, P<0.001)	2.51 (2.20 to 2.87, P<0.001)	2.47 (2.17 to 2.82, P<0.001)
Other or unknown	13 475 (99.6)	48 (0.4)	0.11 (0.08 to 0.15, P<0.001)	0.30 (0.21 to 0.40, P<0.001)	—
Prior emergency department attendances:					
No presentation	365 110 (98.3)	6273 (1.7)	—	—	—
One presentation	54 922 (97.4)	1440 (2.6)	1.53 (1.44 to 1.62, P<0.001)	0.99 (0.93 to 1.06, P=0.808)	0.98 (0.92 to 1.04, P=0.439)
Two presentations	22 729 (96.6)	803 (3.4)	2.06 (1.91 to 2.21, P<0.001)	0.98 (0.90 to 1.07, P=0.666)	0.95 (0.88 to 1.04, P=0.256)
Time of day:					
0600-1159	112 192 (98.1)	2170 (1.9)	—	—	—
1200-1759	159 166 (98.2)	2987 (1.8)	0.97 (0.92 to 1.03, P=0.289)	0.94 (0.89 to 1.00, P=0.045)	0.94 (0.89 to 1.00, P=0.045)
1800-2359	122 051 (98.2)	2260 (1.8)	0.96 (0.90 to 1.02, P=0.151)	0.91 (0.85 to 0.97, P=0.003)	0.91 (0.85 to 0.97, P=0.003)
0000-0559	49 348 (97.8)	1099 (2.2)	1.15 (1.07 to 1.24, P<0.001)	0.96 (0.89 to 1.04, P=0.300)	0.96 (0.89 to 1.04, P=0.288)
Month:					
January to March	106 454 (97.9)	2326 (2.1)	—	—	—
April to June	110 460 (98.2)	2022 (1.8)	0.84 (0.79 to 0.89, P<0.001)	0.91 (0.86 to 0.97, P=0.005)	0.91 (0.86 to 0.97, P=0.005)
July to September	113 062 (98.3)	1922 (1.7)	0.78 (0.73 to 0.83, P<0.001)	0.88 (0.83 to 0.94, P<0.001)	0.88 (0.83 to 0.94, P<0.001)
October to December	112 785 (98.0)	2246 (2.0)	0.91 (0.86 to 0.97, P=0.002)	0.93 (0.87 to 0.99, P=0.024)	0.94 (0.88 to 1.00, P=0.038)
Year:					
2012	51 879 (97.6)	1297 (2.4)	—	—	—
2013	51 309 (97.8)	1165 (2.2)	0.91 (0.84 to 0.98, P=0.018)	1.01 (0.93 to 1.10, P=0.839)	1.02 (0.94 to 1.11, P=0.658)
2014	54 160 (98.1)	1077 (1.9)	0.80 (0.73 to 0.86, P<0.001)	0.80 (0.74 to 0.88, P<0.001)	0.82 (0.75 to 0.90, P<0.001)
2015	52 323 (97.9)	1126 (2.1)	0.86 (0.79 to 0.93, P<0.001)	0.82 (0.75 to 0.89, P<0.001)	0.84 (0.77 to 0.91, P<0.001)
2016	54 447 (98.0)	1095 (2.0)	0.80 (0.74 to 0.87, P<0.001)	0.78 (0.71 to 0.85, P<0.001)	0.80 (0.73 to 0.87, P<0.001)
2017	55 770 (98.3)	988 (1.7)	0.71 (0.65 to 0.77, P<0.001)	0.64 (0.58 to 0.70, P<0.001)	0.67 (0.61 to 0.73, P<0.001)
2018	58 257 (98.4)	928 (1.6)	0.64 (0.59 to 0.69, P<0.001)	0.57 (0.52 to 0.63, P<0.001)	0.60 (0.55 to 0.66, P<0.001)
2019	64 616 (98.7)	840 (1.3)	0.52 (0.48 to 0.57, P<0.001)	0.47 (0.43 to 0.52, P<0.001)	0.50 (0.46 to 0.55, P<0.001)

Logistic regression models (univariable and multivariable) using 30 day mortality as the outcome. No. in dataframe=451 291, No. in model=451 273, missing=18, Akaike Information Criterion=59 075·5, C-statistic=0·923, Hosmer-Lemeshow χ2(degrees of freedom 8)=163·87 (P<0·001). In multiple imputation model, 451 291 patients were included· Missing values for sex (n=14 (0·0%)), Scottish index of multiple deprivation (n=8509 (1·9%)), ethnic group (n=73 646 (16·3%)), triage category (n=13 523 (3·0%)), and presentation time (n=4 (0·0%)) have been imputed using a pool of 10 iterations.

SD, standard deviation.

**Figure 5:**
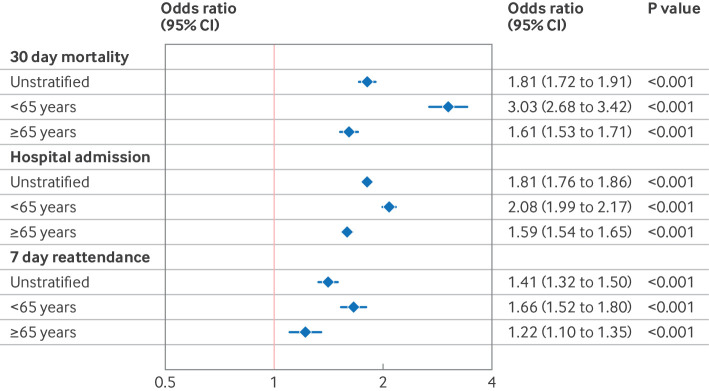
Association between multimorbidity and adverse outcomes, stratified by age category. Unstratified indicates the whole population. Subsequent models are stratified by age category (<65 years *v* ≥65 years). Odds ratios from multivariable logistic regression models for binary outcomes shown (30 day mortality, hospital admission, and seven day reattendance for those discharged home from emergency department alive). Models control for age, sex, ethnic group, Scottish index of multiple deprivation, triage category, previous emergency department attendances, time of day, and month and year. CI=confidence interval

**Figure 6:**
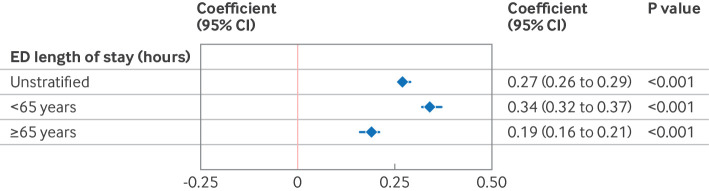
Association between multimorbidity and adverse outcomes, stratified by age category. Unstratified indicates the whole population. Subsequent models are stratified by age category (<65 years *v* ≥65 years). Coefficients from multivariable linear regression models shown for emergency department length of stay (continuous). Models control for age, sex, ethnic group, Scottish index of multiple deprivation, triage category, previous emergency department attendances, time of day, and month and year. CI=confidence interval

After adjustment for other prognostic variables, multimorbidity was associated with increased mortality (adjusted odds ratio 1.81 (95% CI 1.72 to 1.91); Figure 5; Figure 6). In age stratified analyses (figure 4B), risk of death associated with multimorbidity was greater in younger people (<65 years) than in older people (≥65 years) (3.03 (2.68 to 3.42) *v *1.61 (1.53 to 1.71); Figure 5).

The individual long term condition with the highest odds of death was cancer (adjusted odds ratio 2.64 (95% CI 2.48 to 2.81)), followed by drug misuse (2.16 (1.67 to 2.77); efigure 3). When stratified by age, cancer remained the long term condition with the greatest association with death (<65 *v* ≥65 years, 5.67 (4.94 to 6.50) *v* 2.21 (2.06 to 2.36)). Drug misuse was associated with mortality in the younger cohort but not the older cohort (odds ratio 1.94 (95% CI 1.45 to 2.57) *v* 1.08 (0.32 to 2.81); efigures 4A and 4B).

People with multimorbidity were seen more quickly by healthcare professionals in the emergency department than people who did not have multimodity (median 68 *v* 76 mins). Despite this, multimorbidity was associated with longer emergency department length of stay (median 4.0 h (interquartile range 3.2-4.9) *v* 3.3 h (2.3-4.2), adjusted coefficient 0.27 hours (equivalent to 16 min) (95% confidence interval 0.26 to 0.29); table 2; online supplemental eTable 5). Consequently, a lower proportion of people with multimorbidity left the emergency department within 4 hours (49.4% *v* 68.7%). Multimorbidity was more strongly associated with longer emergency department length of stay in the younger patient group (adjusted coefficient 0.34 hours (95% CI 0.32-0.37) *v* 0.19 hours (0.16-0.21); Figure 5).

Admission to hospital after emergency department assessment occurred in 109 750 (24.3%) people. Multimorbidity was associated with higher rates of hospital admission (26 136 (60.1%) people with multimorbidity *v* 83 614 (20.5%) people who do not have multimorbidity, adjusted odds ratio 1.81 (1.76 to 1.86); table 2; online supplemental eTable 6). People with multimorbidity were also more likely to be admitted directly to critical care (1182 (2.7%) *v* 5049 (1.2%)), but less likely to be discharged home (35.0% *v* 68.8%) or to have their care transferred to ambulatory care or outpatient clinics (4.0% *v* 10.4%). Hospital length of stay was longer in people with multimorbidity (4.5 days (interquartile range 1.5-13) *v* 2 days (1-6.5)). Multimorbidity was more strongly associated with hospital admission in the younger group than in the older group (adjusted odds ratio 2.08 (95% CI 1.99 to 2.17) *v* 1.59 (1.54 to 1.65); Figure 5).

Of those discharged from the emergency department alive and were not admitted to hospital, 12 539 (3.7%) reattended an emergency department within seven days. Multimorbidity was associated with higher likelihood of emergency department reattendance within seven days in people with multimorbidity versus people with no multimorbidity (1321 (7.8%) *v* 11 218 (3.5%), adjusted odds ratio 1.41 (95% CI 1.32 to 1.50); table 2; online supplemental eFigure 2A; online supplemental eTable 7; Figure 5). After adjustment, multimorbidity was more strongly associated with seven day reattendance in the younger cohort than in the older cohort (adjusted odds ratio 1.66 (95% CI 1.52 to 1.80) *v* 1.22 (1.10 to 1.35); Figure 5). In an unadjusted cumulative incidence analysis, people younger than 65 years with multimorbidity were more likely to reattend sooner than any other age multimorbidity combination (online supplemental eFigure 2B).

Significant interactions were reported between age and 30 day mortality, emergency department length of stay, hospital admission, and seven day reattendance (etable 8). The age and multimorbidity interaction, when modelled on a continuous scale, showed a diminishing association between multimorbidity and mortality with increasing age (efigure 5). In sensitivity analyses using multiple imputation for all missing data, the associations between multimorbidity and outcomes were largely unchanged (table 3; online supplemental eTables 5-7).

## Discussion

Almost one in ten people attending emergency departments in NHS Lothian were multimorbid, defined using hospital discharge codes for Elixhauser conditions. More older people than younger had multimorbidity, but younger people had a larger burden of psychiatric and substance misuse conditions. People with multimorbidity were older, more likely to live in a deprived area, and had a greater number of previous emergency department presentations. They were more likely to arrive using emergency methods of transport and to be more severely ill on presentation. Multimorbidity was strongly associated with mortality, length of time spent in the emergency department, hospital admission, and reattendance to the emergency department. Furthermore, the associations between multimorbidity and all outcomes were of greater magnitude in younger attendees of the emergency department compared with older attendees.

Previous studies reported a range of estimates for multimorbidity prevalence in UK populations, ranging from 11% to 42%.^14 24^,^26^ These estimates are much higher than in our study. As we derived only Elixhauser conditions from hospital discharge records in the five years prior to attendance, the prevalence of multimorbidity in this study is likely to be an underestimate compared with other studies that used a greater number of conditions^24^ or primary care diagnoses^1 4 25^ for ascertainment. The impact of this on the associations between multimorbidity and outcomes is unclear because long term conditions derived from hospital records, rather than primary care records, may lead to associations of greater magnitude.

Our study provides a useful addition to existing knowledge. This is the largest study investigating multimorbidity in the context of emergency medicine services. Our findings are broadly consistent with findings from a recent much smaller study focusing on emergency department attendees in Glasgow, Scotland.^26^ The authors found that multimorbidity among people attending emergency department was strongly associated with hospital admission and emergency department reattendance, but was not associated with mortality. However, multimorbidity was defined using a different set of conditions, and a shorter look-back period of 21 months before the index emergency department attendance. Length of stay in emergency department was not reported, no comparison was available for assessing the interaction between outcomes and age, and the association between different comorbidities and mortality was not investigated.

Our finding that multimorbidity prevalence in emergency department populations increases with age and deprivation, and is associated with increased mortality, supports the findings in studies undertaken in general population based cohorts.^1 4 24^ Although literature concerning multimorbidity in emergency department attendees is scarce, in general population based studies, multimorbidity has been associated with higher emergency department attendance^27^ and hospital admission.^4 28^

The larger association between multimorbidity and mortality in a younger cohort compared with an older cohort has previously been reported in general population based studies.^24 29^ In a Northern Irish population based study of younger people (age 25-64 years), social determinants were strongly associated with multimorbidity and the most disadvantaged groups were at a high risk of physical and mental health multimorbidity.^30^ An English population based study investigated the association between different multimorbidity clusters, mortality, and service use.^4^ The long term condition cluster associated with the highest mortality in a younger age group comprised substance misuse in combination with alcohol problems. These studies' findings accord with our own in that substance misuse and psychiatric long term conditions were more common in the under 65s, and more strongly associated with mortality than in an older cohort.

Our study describes multimorbidity in an emergency department population, rather than in populations defined by primary care records or in the general population. Our results have strength from linkage of a range of data sources over eight years to systematically describe multimorbidity and analyse a large population with precise results. Additionally, our analysis benefits from examining processes within emergency department, such as time spent in department and time to be seen by a healthcare provider, which can inform health services when planning processes surrounding the care of people with multimorbidity in their emergency departments.

Limitations include the sourcing of data from a single territorial health board in Scotland, which might affect generalisability outside of this context. However, with very few inclusion and exclusion criteria, as well as complete capture of all emergency department attendances within NHS Lothian, findings are likely to be generalisable to UK and other, similar healthcare systems. Additionally, our data pre-date the onset of the covid-19 pandemic and the subsequent difficulties facing hospitals in the UK and worldwide. While our results are unaffected by the changes to hospital and emergency department working patterns that occurred during or because of the pandemic, they may now be difficult to generalise to departments that have experienced large changes. We were unable to account for clustering at hospital level, which may lead to overly precise estimates. Furthermore, important prognostic factors may not be accounted for in regression models including data related to severity of illness on presentation other than triage category, pre-admission social care needs, and household composition, which affect likelihood of hospital admission and emergency department reattendance.

A further limitation is that long term conditions and therefore multimorbidity were ascertained only from hospital discharge data in the five years prior to emergency department attendance, and only for conditions included in the Elixhauser index without taking into account long term condition severity. Analysis may therefore only capture more severe long term conditions and so underestimate the prevalence of multimorbidity by as much as 50%.^31^ However, long term conditions derived from hospital data have stronger associations with adverse outcomes than those derived from primary care data.^31^

We have identified that associations between multimorbidity and poor outcomes are larger in younger emergency department attendees compared with older attendees. This may, in part, be explained by a difference in the types of conditions present in the younger cohort. Another factor could be that multimorbidity may be better recognised in older patient groups, in part due to the overlap between older age, frailty, and multimorbidity. Some older people with multimorbidity may be identified by frailty teams or other appropriate services, which have expertise in managing emergency presentations in the context of multiple long term conditions. By contrast, the younger population will likely have multimorbidity that is not identified by these services and therefore receive less well integrated care.^32^

Many studies suggest adoption of a consensus defined definition of multimorbidity and the comprising conditions.^2533^,^35^ Such consensus methods may need to be modified to identify long term conditions of more relevance to the emergency department setting. Extending our study with data following the covid-19 pandemic may also add further insights to the impact of multimorbidity in contemporary emergency departments. Further research is also needed on defining different condition clusters, and how these may affect people at different stages of their lives.

Our study supports the notion that multimorbidity is an important factor in people presenting to the emergency department due to the associated range of adverse patient centred outcomes. Mechanisms for this might include the complexities of treating people with multimorbidity using conventional single organ protocols and care pathways and a higher risk of adverse events in this population.^36^ ^37^ The conditions that cause people to have multimorbidity could also predispose them to be more severely ill at presentation.

The increasing prevalence of multimorbidity in the population is likely to exacerbate strain on emergency departments unless practice and policy evolve to meet the growing demand. Our study therefore has important implications for clinical practice. Our findings support the need to better recognise people in emergency department settings who may benefit from tailored care that accounts for multimorbidity, which may be challenging for clinicians who are time limited and often have limited previous knowledge of the individuals that they are treating. This may include the use of multidisciplinary teams in the acute care setting, which may help to identify and care for people who would benefit from multi-specialty involvement.^38^ Given the high mortality in the cohort of people with multimorbidity, identification of multimorbidity may help act as a prompt to establish treatment goals with people and families at an early timepoint during their acute illness. Establishing patient values and treatment preferences can help to ensure care is aligned with preferences, and is a key part of realistic medicine, a policy by the Scottish Government aimed at empowering people to have more holistic, preference aligned healthcare.^39^

Our research adds weight to previous literature findings that social deprivation, substance misuse, and mental health conditions are drivers of multimorbidity and worse outcomes, especially in younger people.^4 30^ Our findings have implications for key areas of policy change to help improve health outcomes and quality of life, especially given the high level of drug related deaths within the Scottish population.^40^

## Conclusion

In this population based cohort study, almost one in ten people presenting to emergency department had multimorbidity. Multimorbidity was strongly associated with adverse outcomes, and these associations were more pronounced in younger people. People with multimorbidity in emergency department settings might benefit from improved recognition and tailored care pathways.

## Supplementary material

10.1136/bmjmed-2023-000731 online supplemental file 1

10.1136/bmjmed-2023-000731 online supplemental file 2

## Data Availability

No data are available.
