# Supplementary material for: Multimorbidity and adverse outcomes following emergency department attendance: population based cohort study
Source: BMJ Med. 2024 Aug 16;3(1):e000731. doi: 10.1136/bmjmed-2023-000731 (PMC11344864; doi:10.1136/bmjmed-2023-000731)

Supplementary materials

Multimorbidity and adverse outcomes following Emergency Department attendance: a cohort study

Michael Blayney[1] (MBChB), Matthew Reed[2] (MD), John Masterson[1] (BMedSci(Hons)), Atul Anand[3] (PhD), Matt Bouamrane[4] (PhD), Saturnino Luz Filho[4] (PhD), Jacques Fleuriot[5,7] (PhD), Marcus Lyall[6] (PhD), Stewart Mercer[2,5] (PhD), Nicholas Mills[2,3] (PhD), Susan Shenkin[2] (MD), Tim Walsh[1,6] (MD), Sarah Wild[2] (PhD), Honghan Wu[8,9] (PhD), Stela McLachlan[2,4] (PhD), Bruce Guthrie[2,5] (PhD), Nazir Lone[1,2,6] (PhD)

1 Dept of Anaesthesia, Critical Care and Pain Medicine, Usher Institute, University of Edinburgh, Edinburgh, EH16 4SA, UK
2 Centre for Population Health Sciences, Usher Institute, University of Edinburgh, Teviot Place, Edinburgh, EH8 9AG, UK
3 Centre for Cardiovascular Science, University of Edinburgh, 49 Little France Crescent, Edinburgh, EH16 4SB
4 Centre for Medical Informatics, Usher Institute, University of Edinburgh, Teviot Place, Edinburgh, EH8 9AG, UK
5 Advanced Care Research Centre, Usher Institute, University of Edinburgh, BioCube 1, BioQuarter, 13 Little France, Edinburgh, EH16 4UX, UK
6 Royal Infirmary of Edinburgh, NHS Lothian, 51 Little France Crescent, Edinburgh, EH16 4SA, UK
7 Artificial Intelligence and its Applications Institute, School of Informatics, University of Edinburgh, EH8 9AB, UK
8 Institute of Health Informatics, University College London, 222 Euston Road, London, NW1 2DA
9 The Alan Turing Institute, British Library, 96 Euston Rd., London NW1 2DB

eTable 1: Long-term condition definitions

Long-term condition	Elixhauser Category	ICD-10 codes
Hypertension	Hypertension without complications	I10
	Hypertension with complications	I11, I12, I13, I15
Chronic pulmonary disease	Chronic pulmonary disease	I278, I279, J40, J41, J42, J43, J44, J45, J46, J47, J60, J61, J62, J63, J64, J65, J66, J67, J684, J701, J703
Cancer	Solid tumour without metastases	C00, C01, C02, C03, C04, C05, C06, C07, C08, C09, C10, C11, C12, C13, C14, C15, C16, C17, C18, C19, C20, C21, C22, C23, C24, C25, C26, C30, C31, C32, C33, C34, C37, C38, C39, C40, C41, C43, C45, C46, C47, C48, C49, C50, C51, C52, C53, C54, C55, C56, C57, C58, C60, C61, C62, C63, C64, C65, C66, C67, C69, C70, C71, C72, C73, C74, C75, C76, C97
	Solid tumour with metastases	C77, C78, C79, C80
	Lymphoma	C81, C82, C83, C84, C85, C88, C96, C900, C902
Cardiac Arrhythmia	Cardiac arrhythmia	I441, I442, I443, I456, I47, I48, I49, R000, R008, T821, Z450, Z950
Diabetes	Diabetes without chronic complications	E100, E101, E109, E110, E111, E119, E120, E121, E129, E130, E131, E139, E140, E141, E149
	Diabetes with chronic complications	E102, E103, E104, E105, E106, E107, E108, E112, E113, E114, E115, E116, E117, E118, E122, E123, E124, E125, E126, E127, E128, E132, E133, E134, E135, E136, E137, E138, E142, E143, E144, E145, E146, E147, E148
Alcohol dependence	Alcohol abuse	F10, E52, G621, I426, K292, K700, K703, K709, T51, Z502, Z714, Z721
Fluid and electrolyte disorders	Fluid and electrolyte disorders	E222, E86, E87
Congestive Heart Failure	Congestive heart failure	I099, I110, I130, I132, I255, I420, I425, I426, I427, I428, I429, I43, I50, P290
Renal failure	Renal failure	I120, I131, N18, N19, N250, Z490, Z491, Z492, Z940, Z992
Other neurological disorder	Other neurological disorder	G10, G11, G12, G13, G20, G21, G22, G254, G255, G312, G318, G319, G32, G35, G36, G37, G40, G41, G931, G934, R470, R56
Valvular heart disease	Valvular heart disease	A520, I05, I06, I07, I08, I019, I098, I34, I35, I36, I37, I38, I39, Q230, Q231, Q232, Q233, Z952, Z953, Z954
Peripheral Vascular disease	Peripheral vascular disease	I70, I71, I731, I738, I739, I771, I790, I792, K551, K558, K559, Z958, Z959
Depression	Depression	F204, F313, F314, F315, F32, F33, F341, F412, F432
Deficiency anaemia	Deficiency anaemia	D508, D509, D51, D52, D53
Liver disease	Liver disease	B18, I85, I864, I982, K70, K711, K713, K714, K715, K717, K72, K73, K74, K760, K762, K763, K764, K765, K766, K767, K768, K769, Z944
Drug misuse	Drug abuse	F11, F12, F13, F14, F15, F16, F18, F19, Z715, Z722
Rheumatological/Collagen disease	Rheumatological/Collagen disorder	L940, L941, L943, M05, M06, M08, M120, M123, M30, M310, M311, M312, M313, M32, M33, M34, M35, M45, M461, M468, M469
Diseases of pulmonary circulation	Diseases of pulmonary circulation	I26, I27, I280, I288, I289
Hypothyroidism	Hypothyroidism	E00, E01, E02, E03, E890
Obesity	Obesity	E66
Psychoses	Psychoses	F20, F22, F23, F24, F25, F28, F29, F302, F12, F315
Weight loss	Weight loss	E40, E41, E41, E42, E43, E45, E46, R634, R64
Diseases of coagulation	Diseases of coagulation	D65, D66, D67, D68, D691, D693, D694, D695, D696
Paralysis	Paralysis	G041, G114, G801, G802, G81, G82, G830, G831, G833, G834, G839
Peptic ulcer disease (without bleeding)	Peptic ulcer disease (without bleeding)	K257, K259, K267, K269, K277, K279, K287, K289
AIDS/HIV	AIDS/HIV	B20, B21, B22, B24
Blood loss anaemia	Blood loss anaemia	D500

eTable 2: Variable definitions (outcomes)

Outcome	Category	Definition	Data source
Departmental Outcomes			
Time (minutes) to be seen		Time between person presenting in ED and first seen by a Healthcare provider	EDTrak
Seen by HCP within 4 hours	No<br>Yes	Person seen by a Healthcare Provider within 4 hours of presentation (a key ED management goal in the UK)	EDTrak
Hours spent in ED	Median (IQR)<br>Mean (SD)	Time between person presenting in ED and leaving ED (via discharge, hospital admission or death)	EDTrak
Left ED within 4 hrs	No<br>Yes	Person leaving ED within 4 hours of presentation (a key ED management goal in the UK)	EDTrak
Discharge Outcomes			
Discharge destination	Admission - Critical Care or CCU	Admission to critical care (intensive care unit or high dependency unit) or coronary care unit	EDTrak
	Admission to Hospital Ward<br>Died before hospital admission<br>Home<br>Outpatient clinic/Ambulatory Care		
Level of care admitted to	CCU<br>HDU<br>ICU<br>non-CC<br>Not admitted	Coronary care unit<br>High Dependency Unit (Level 2 area)<br>Intensive Care Unit (Level 3 area)<br>Non-critical care ward<br>Not admitted to hospital	EDTrak
Hospital LOS (days)	Median (IQR)<br>Mean (SD)	Time between hospital admission (immediately following index ED attendance) and discharge, for those admitted to hospital	SMR01
Repeat ED presentation within 7 days	No<br>Yes	Person reattended ED within 7 days of index ED attendance (for the cohort of people discharged from ED alive – ie did not die in the department, and were not admitted to hospital)	EDTrak
Mortality Outcomes			
Died within 24hrs of presentation	Died<br>Survived		NRS Death Records
Died within 7 days of presentation	Died<br>Survived		NRS Death Records
Died within 30 days of presentation	Died<br>Survived		NRS Death Records
In-hospital mortality	Died<br>Survived	Mortality for patients admitted to hospital following their index ED attendance	NRS Death Records and SMR01

eTable 3: Variable definitions (excluding outcomes)

Variables	Categories	Definition	Data Source
Multimorbidity*	Not multimorbid<br>Multimorbid	1 or 0 LTCs in hospital records in 5 years prior to ED attendance<br>2 or more LTCs in hospital records in 5 years prior to ED attendance	SMR01 and SMR06
Age*		Age at ED attendance	EDTrak
Sex*	Female<br>Male		EDTrak
Ethnicity*	White<br>Asian<br>African/Black<br>Other/Mixed<br>Refused/Unknown/Missing	Self-reported ethnicity	EDTrak
SIMD*	5 - least deprived<br>4<br>3<br>2<br>1 - most deprived<br>SIMD Unknown	Scottish Index of Multiple Deprivation – deprivation score linked to person's residential postcode. Divided into quintiles throughout Scotland	EDTrak
Illness severity			
Referral	999 Emergency<br>Flow Centre<br>GP<br><br>NHS24<br><br>Other<br>Self-referral<br>Unscheduled Care Services	People attending ED following a "999" call to emergency services<br>People attending ED from another secondary healthcare setting<br>People attending ED following contacting their GP (but not necessarily after being assessed by a GP)<br>People attending ED following a call to NHS24 (less urgent than "999")<br>Other referral category<br>People attending ED without referral from other health services<br>People attending ED after contacting and/or being assessed by out of hours general medical services (for example, Out of Hours GP assessment)	EDTrak
Arrival Mode	Emergency Ambulance<br>Urgent Ambulance<br>Private Transport<br>Public Transport<br>Other	People arriving in an emergency ambulance<br>People arriving in an urgent ambulance<br>People arriving using own/private transport<br>People arriving using public transport<br>People arriving using other transport	EDTrak
Triage category*	Medical expected<br>See and treat<br><br>Standard or Non-urgent<br>Urgent<br>Very urgent or immediate<br><br>Other/Unknown	People assessed by a GP and advised to attend ED<br>People whose conditions are expected to be treatable in the department without hospital admission<br>People requiring medical attention but in a non-urgent fashion<br>People requiring urgent medical attention<br>People whose life or limb-threatening condition is requiring very urgent/immediate medical attention<br>Triage category was missing or unable to be categorised	EDTrak
Previous health status			
ED attendances*	0 in previous year<br>1 in previous year<br>2 or more in previous year	Number of ED attendances to NHS Lothian in the 365 days prior to index ED attendance	EDTrak – derived
Hospital admissions	0 in previous year<br>1 in previous year<br>2 or more in previous year	Number of hospital admissions to any hospitals in Scotland in the 365 days prior to index ED attendance	SMR01
Time of day*	0600-1159<br>1200-1759<br>1800-2359<br>0000-0559	Time of presentation to ED	EDTrak
Month*	January to March<br>April to June<br>July to September<br>October to December	Month of presentation to ED	EDTrak
Year*	2012<br>2013<br>2014<br>2015<br>2016<br>2017<br>2018<br>2019	Year of presentation to ED	EDTrak

* Indicates terms entered into regression modelling.

eTable 4: Long-term condition prevalence, stratified by age group

LTC	All	<65 years	≥65 years
n	451291	342289	109002
Hypertension	21496 (4·8%)	4907 (1·4%)	16589 (15·2%)
Chronic pulmonary disease	17232 (3·8%)	6429 (1·9%)	10803 (9·9%)
Cancer	15842 (3·5%)	5118 (1·5%)	10724 (9·8%)
Cardiac arrhythmia	15701 (3·5%)	2667 (0·8%)	13034 (12·0%)
Diabetes	12780 (2·8%)	4370 (1·3%)	8410 (7·7%)
Alcohol abuse	10568 (2·3%)	8371 (2·4%)	2197 (2·0%)
Fluid and electrolyte disorders	7511 (1·7%)	2059 (0·6%)	5452 (5·0%)
Congestive heart failure	7436 (1·6%)	1121 (0·3%)	6315 (5·8%)
Renal failure	7420 (1·6%)	1102 (0·3%)	6318 (5·8%)
Other neurological disorder	6574 (1·5%)	3477 (1·0%)	3097 (2·8%)
Valvular heart disease	6081 (1·3%)	1015 (0·3%)	5066 (4·6%)
Peripheral vascular disease	5251 (1·2%)	1074 (0·3%)	4177 (3·8%)
Depression	4761 (1·1%)	3315 (1·0%)	1446 (1·3%)
Deficiency anaemia	4454 (1·0%)	1132 (0·3%)	3322 (3·0%)
Liver disease	4306 (1·0%)	2581 (0·8%)	1725 (1·6%)
Drug misuse	3748 (0·8%)	3691 (1·1%)	57 (0·1%)
Rheumatological/collagen disease	3273 (0·7%)	1053 (0·3%)	2220 (2·0%)
Diseases of pulmonary circulation	3147 (0·7%)	1001 (0·3%)	2146 (2·0%)
Hypothyroidism	3087 (0·7%)	841 (0·2%)	2246 (2·1%)
Obesity	2803 (0·6%)	1680 (0·5%)	1123 (1·0%)
Psychoses	2430 (0·5%)	1966 (0·6%)	464 (0·4%)
Weight loss	1742 (0·4%)	638 (0·2%)	1104 (1·0%)
Diseases of coagulation	1543 (0·3%)	807 (0·2%)	736 (0·7%)
Paralysis	1037 (0·2%)	457 (0·1%)	580 (0·5%)
Peptic ulcer disease (without bleeding)	874 (0·2%)	352 (0·1%)	522 (0·5%)
AIDS/HIV	193 (0·0%)	182 (0·1%)	11 (0·0%)
Blood loss anaemia	137 (0·0%)	56 (0·0%)	81 (0·1%)

eTable 5: Linear regression model – Length of stay in the emergency department

Outcome: ED Length of Stay (hours)		Mean (SD) Length of stay (hours)	Coefficient (95% CI) (univariable)	Coefficient (95% CI) (multivariable)	Coefficient (95% CI) (multiple imputation)
Multimorbidity	No multimorbidity	3·3 (1·6)	-	-	-
	Multimorbidity	4·3 (1·9)	0·91 (0·89 - 0·92) p<0·001	0·27 (0·26 - 0·29) p<0·001	0·28 (0·27-0·30), p<0·001
Age	Per 1 year increase	3·4 (1·6)	0·02 (0·02 - 0·02), p<0·001	0·01 (0·01 - 0·01), p<0·001	0·01 (0·01-0·01), p<0·001
Sex	Women	3·5 (1·6)	-	-	-
	Men	3·4 (1·6)	-0·04 (-0·05 - -0·03), p<0·001	0·01 (0·00 - 0·02), p=0·009	0·01 (0·01-0·02), p=0·001
Ethnicity	White	3·4 (1·6)	-	-	-
	Asian	3·2 (1·6)	-0·24 (-0·27 - -0·20), p<0·001	-0·06 (-0·09 - -0·03), p<0·001	-0·11 (-0·14--0·08), p<0·001
	African/Black	3·2 (1·6)	-0·20 (-0·26 - -0·14), p<0·001	-0·02 (-0·07 - 0·03), p=0·403	-0·05 (-0·11-0·00), p=0·066
	Other/Mixed	3·1 (1·5)	-0·38 (-0·40 - -0·35), p<0·001	-0·08 (-0·11 - -0·06), p<0·001	-0·14 (-0·16--0·12), p<0·001
	Refused/Unknown/ Missing	3·5 (1·7)	0·05 (0·04 - 0·07), p<0·001	-0·00 (-0·01 - 0·01), p=0·720	-
SIMD quintile	5 - least deprived	3·5 (1·7)	-	-	-
	4	3·4 (1·6)	-0·06 (-0·07 - -0·04), p<0·001	-0·02 (-0·03 - -0·00), p=0·009	-0·02 (-0·03--0·00), p=0·014
	3	3·4 (1·6)	-0·03 (-0·05 - -0·02), p<0·001	-0·01 (-0·02 - 0·01), p=0·335	-0·01 (-0·02-0·00), p=0·203
	2	3·4 (1·6)	-0·00 (-0·02 - 0·01), p=0·554	-0·01 (-0·02 - 0·00), p=0·126	-0·01 (-0·02-0·00), p=0·058
	1 - most deprived	3·4 (1·6)	-0·02 (-0·03 - -0·00), p=0·049	-0·00 (-0·02 - 0·01), p=0·563	-0·01 (-0·02-0·00), p=0·158
	SIMD Unknown	3·2 (1·6)	-0·21 (-0·25 - -0·18), p<0·001	-0·02 (-0·05 - 0·01), p=0·150	-
ED attendances	0 in previous year	3·4 (1·6)	-	-	-
	1 in previous year	3·6 (1·7)	0·18 (0·16 - 0·19), p<0·001	0·05 (0·03 - 0·06), p<0·001	0·04 (0·03-0·05), p<0·001
	2 or more in previous year	3·8 (1·8)	0·43 (0·40 - 0·45), p<0·001	0·08 (0·06 - 0·10), p<0·001	0·07 (0·05-0·09), p<0·001
Triage category	Medical expected	3·9 (1·7)	-	-	-
	See and treat	2·5 (1·1)	-1·35 (-1·38 - -1·31), p<0·001	-0·94 (-0·97 - -0·90), p<0·001	-0·97 (-1·00--0·93), p<0·001
	Standard or Non-urgent	3·1 (1·3)	-0·79 (-0·82 - -0·75), p<0·001	-0·40 (-0·43 - -0·36), p<0·001	-0·40 (-0·44--0·37), p<0·001
	Urgent	3·8 (1·7)	-0·07 (-0·10 - -0·04), p<0·001	0·20 (0·17 - 0·23), p<0·001	0·21 (0·18-0·24), p<0·001
	Very urgent or immediate	4·3 (1·8)	0·36 (0·33 - 0·40), p<0·001	0·47 (0·44 - 0·50), p<0·001	0·48 (0·44-0·51), p<0·001
	Other/Unknown	1·6 (1·1)	-2·27 (-2·32 - -2·23), p<0·001	-1·88 (-1·92 - -1·85), p<0·001	-
Time of day	0600-1159	3·1 (1·5)	-	-	-
	1200-1759	3·4 (1·5)	0·27 (0·26 - 0·29), p<0·001	0·26 (0·25 - 0·28), p<0·001	0·26 (0·25-0·27), p<0·001
	1800-2359	3·6 (1·7)	0·46 (0·45 - 0·47), p<0·001	0·44 (0·43 - 0·45), p<0·001	0·42 (0·41-0·43), p<0·001
	0000-0559	3·7 (2·0)	0·61 (0·59 - 0·63), p<0·001	0·47 (0·46 - 0·49), p<0·001	0·46 (0·45-0·48), p<0·001
Month	January - March	3·0 (1·7)	-	-	-
	April - June	3·8 (1·5)	0·82 (0·81 - 0·83), p<0·001	0·86 (0·85 - 0·87), p<0·001	0·85 (0·84-0·87), p<0·001
	July - September	3·7 (1·4)	0·77 (0·76 - 0·78), p<0·001	0·83 (0·82 - 0·84), p<0·001	0·83 (0·81-0·84), p<0·001
	October - December	3·2 (1·7)	0·21 (0·20 - 0·22), p<0·001	0·22 (0·21 - 0·23), p<0·001	0·22 (0·21-0·23), p<0·001
Year	2012	3·3 (1·6)	-	-	-
	2013	3·2 (1·6)	-0·10 (-0·12 - -0·08), p<0·001	-0·07 (-0·08 - -0·05), p<0·001	-0·06 (-0·08--0·04), p<0·001
	2014	3·2 (1·5)	-0·11 (-0·13 - -0·09), p<0·001	-0·12 (-0·13 - -0·10), p<0·001	-0·10 (-0·12--0·09), p<0·001
	2015	3·3 (1·5)	0·01 (-0·01 - 0·03), p=0·163	-0·00 (-0·02 - 0·02), p=0·896	0·01 (-0·01-0·02), p=0·466
	2016	3·5 (1·5)	0·13 (0·12 - 0·15), p<0·001	0·11 (0·10 - 0·13), p<0·001	0·12 (0·10-0·14), p<0·001
	2017	3·5 (1·6)	0·21 (0·19 - 0·23), p<0·001	0·16 (0·14 - 0·18), p<0·001	0·17 (0·15-0·19), p<0·001
	2018	3·7 (1·9)	0·37 (0·36 - 0·39), p<0·001	0·36 (0·35 - 0·38), p<0·001	0·38 (0·36-0·39), p<0·001
	2019	3·6 (1·8)	0·24 (0·22 - 0·25), p<0·001	0·23 (0·21 - 0·25), p<0·001	0·25 (0·23-0·27), p<0·001

eTable 6: Logistic regression model – Hospital Admission.

Outcome: Hospital Admission		Not admitted No. (%)	Admitted No. (%)	OR (95% CI) (univariable)	OR (95% CI) (multivariable)	OR (95% CI) (multiple imputation)
Multimorbidity	No multimorbidity	324173 (79·5)	83614 (20·5)	-	-	-
	Multimorbidity	17368 (39·9)	26136 (60·1)	5·83 (5·72-5·96, p<0·001)	1·81 (1·76-1·86, p<0·001)	1·82 (1·78-1·87, p<0·001)
Age	Mean (SD)	43·2 (18·7)	61·8 (20·9)	1·05 (1·05-1·05, p<0·001)	1·03 (1·03-1·03, p<0·001)	1·03 (1·03-1·03, p<0·001)
Sex	Women	169240 (74·9)	56653 (25·1)	-	-	-
	Men	172291 (76·4)	53093 (23·6)	0·92 (0·91-0·93, p<0·001)	0·96 (0·95-0·98, p<0·001)	0·96 (0·94-0·98, p<0·001)
Ethnicity	White	263219 (74·9)	88279 (25·1)	-	-	-
	Asian	7351 (84·1)	1388 (15·9)	0·56 (0·53-0·60, p<0·001)	0·83 (0·78-0·89, p<0·001)	0·80 (0·75-0·86, p<0·001)
	African/Black	2442 (84·5)	448 (15·5)	0·55 (0·49-0·60, p<0·001)	0·85 (0·75-0·95, p=0·004)	0·81 (0·72-0·92, p=0·001)
	Other/Mixed	12834 (88·4)	1684 (11·6)	0·39 (0·37-0·41, p<0·001)	0·75 (0·71-0·80, p<0·001)	0·73 (0·69-0·77, p<0·001)
	Refused/Unknown/Missing	55695 (75·6)	17951 (24·4)	0·96 (0·94-0·98, p<0·001)	0·99 (0·96-1·01, p=0·225)	-
SIMD	5 - least deprived	86343 (75·7)	27791 (24·3)	-	-	-
	4	63367 (76·7)	19229 (23·3)	0·94 (0·92-0·96, p<0·001)	1·07 (1·04-1·10, p<0·001)	1·07 (1·04-1·10, p<0·001)
	3	61250 (75·7)	19692 (24·3)	1·00 (0·98-1·02, p=0·916)	1·11 (1·08-1·14, p<0·001)	1·11 (1·08-1·14, p<0·001)
	2	75800 (74·5)	25936 (25·5)	1·06 (1·04-1·08, p<0·001)	1·12 (1·10-1·15, p<0·001)	1·12 (1·09-1·15, p<0·001)
	1 - most deprived	47593 (75·1)	15781 (24·9)	1·03 (1·01-1·05, p=0·010)	1·17 (1·13-1·20, p<0·001)	1·16 (1·13-1·19, p<0·001)
	SIMD Unknown	7188 (84·5)	1321 (15·5)	0·57 (0·54-0·61, p<0·001)	0·92 (0·86-0·99, p=0·027)	-
ED attendances	0 in previous year	287662 (77·5)	83729 (22·5)	-	-	-
	1 in previous year	39473 (70·0)	16890 (30·0)	1·47 (1·44-1·50, p<0·001)	1·17 (1·14-1·20, p<0·001)	1·17 (1·14-1·20, p<0·001)
	2 or more in previous year	14406 (61·2)	9131 (38·8)	2·18 (2·12-2·24, p<0·001)	1·25 (1·20-1·29, p<0·001)	1·25 (1·21-1·30, p<0·001)
Triage category	Medical expected	3413 (40·0)	5109 (60·0)	-	-	-
	See and treat	66783 (99·4)	374 (0·6)	0·00 (0·00-0·00, p<0·001)	0·01 (0·01-0·01, p<0·001)	0·01 (0·01-0·01, p<0·001)
	Standard or Non-urgent	123007 (95·1)	6313 (4·9)	0·03 (0·03-0·04, p<0·001)	0·04 (0·04-0·05, p<0·001)	0·04 (0·04-0·05, p<0·001)
	Urgent	113069 (68·5)	52061 (31·5)	0·31 (0·29-0·32, p<0·001)	0·34 (0·32-0·36, p<0·001)	0·34 (0·32-0·36, p<0·001)
	Very urgent or immediate	22290 (33·0)	45349 (67·0)	1·36 (1·30-1·42, p<0·001)	1·22 (1·16-1·28, p<0·001)	1·22 (1·16-1·29, p<0·001)
	Other/Unknown	12979 (96·0)	544 (4·0)	0·03 (0·03-0·03, p<0·001)	0·04 (0·04-0·05, p<0·001)	-
Time of day	0600-1159	90010 (78·7)	24354 (21·3)	-	-	-
	1200-1759	123853 (76·4)	38301 (23·6)	1·14 (1·12-1·16, p<0·001)	1·08 (1·05-1·10, p<0·001)	1·07 (1·05-1·10, p<0·001)
	1800-2359	92865 (74·7)	31453 (25·3)	1·25 (1·23-1·28, p<0·001)	1·23 (1·20-1·26, p<0·001)	1·22 (1·19-1·25, p<0·001)
	0000-0559	34809 (69·0)	15642 (31·0)	1·66 (1·62-1·70, p<0·001)	1·41 (1·37-1·45, p<0·001)	1·41 (1·37-1·45, p<0·001)
Month	January - March	81174 (74·6)	27609 (25·4)	-	-	-
	April - June	85877 (76·3)	26609 (23·7)	0·91 (0·89-0·93, p<0·001)	0·97 (0·95-1·00, p=0·039)	0·97 (0·95-1·00, p=0·021)
	July - September	88239 (76·7)	26748 (23·3)	0·89 (0·87-0·91, p<0·001)	0·99 (0·97-1·01, p=0·392)	0·99 (0·96-1·01, p=0·226)
	October - December	86251 (75·0)	28784 (25·0)	0·98 (0·96-1·00, p=0·051)	0·99 (0·97-1·02, p=0·629)	0·99 (0·97-1·02, p=0·638)
Year	2012	39706 (74·7)	13470 (25·3)	-	-	-
	2013	39785 (75·8)	12689 (24·2)	0·94 (0·91-0·97, p<0·001)	1·00 (0·97-1·04, p=0·844)	1·01 (0·97-1·04, p=0·714)
	2014	41100 (74·4)	14138 (25·6)	1·01 (0·99-1·04, p=0·319)	1·06 (1·02-1·10, p=0·001)	1·06 (1·03-1·10, p=0·001)
	2015	40130 (75·1)	13320 (24·9)	0·98 (0·95-1·01, p=0·122)	0·96 (0·92-0·99, p=0·011)	0·96 (0·92-0·99, p=0·011)
	2016	42942 (77·3)	12604 (22·7)	0·87 (0·84-0·89, p<0·001)	0·77 (0·75-0·80, p<0·001)	0·77 (0·75-0·80, p<0·001)
	2017	43249 (76·2)	13510 (23·8)	0·92 (0·90-0·95, p<0·001)	0·76 (0·73-0·79, p<0·001)	0·76 (0·74-0·79, p<0·001)
	2018	45523 (76·9)	13665 (23·1)	0·88 (0·86-0·91, p<0·001)	0·70 (0·68-0·73, p<0·001)	0·71 (0·68-0·73, p<0·001)
	2019	49106 (75·0)	16354 (25·0)	0·98 (0·96-1·01, p=0·170)	0·81 (0·78-0·84, p<0·001)	0·82 (0·79-0·84, p<0·001)

Logistic regression models (univariable and multivariable) using hospital admission as the outcome· Number in dataframe = 451291, Number in model = 451273, Missing = 18, AIC = 328262·1, C-statistic = 0·877, H&L = Chi-sq(8) 130·37 (p<0·001). 451291 patients were included in the multiple imputation model. Missing values for Sex (n=14 (0%)), SIMD (n=8509 (1·9%)), Ethnicity (n=73646 (16·3%)), Triage category (n=13523 (3%)), and presentation time (n=4 (0%)) have been imputed using a pool of 10 iterations.

eTable 7: Logistic regression model – 7-day reattendance.

Outcome: Reattendance within 7 days		Did not reattend No. (%)	Reattended No. (%)	OR (95%CI) (univariable)	OR (95% CI) (multivariable)	OR (95%CI) (multiple imputation)
Multimorbidity	No multimorbidity	309773 (96·5)	11218 (3·5)	-	-	-
	Multimorbidity	15553 (92·2)	1321 (7·8)	2·35 (2·21-2·49, p<0·001)	1·41 (1·32-1·50, p<0·001)	1·42 (1·33-1·52, p<0·001)
Age	Mean (SD)	43·0 (18·6)	45·9 (19·6)	1·01 (1·01-1·01, p<0·001)	1·01 (1·01-1·01, p<0·001)	1·01 (1·00-1·01, p<0·001)
Sex	Female	161673 (96·3)	6184 (3·7)	-	-	-
	Male	163643 (96·3)	6355 (3·7)	1·02 (0·98-1·05, p=0·405)	1·04 (1·00-1·08, p=0·026)	1·04 (1·00-1·08, p=0·036)
Ethnicity	White	250147 (96·1)	10202 (3·9)	-	-	-
	Asian	7041 (96·5)	252 (3·5)	0·88 (0·77-0·99, p=0·044)	0·93 (0·81-1·05, p=0·250)	0·93 (0·82-1·06, p=0·265)
	African/Black	2358 (97·2)	69 (2·8)	0·72 (0·56-0·90, p=0·007)	0·73 (0·57-0·92, p=0·009)	0·72 (0·58-0·91, p=0·006)
	Other/Mixed	12254 (96·3)	469 (3·7)	0·94 (0·85-1·03, p=0·187)	1·03 (0·93-1·13, p=0·602)	1·03 (0·94-1·14, p=0·470)
	Refused/Unknown/Missing	53526 (97·2)	1547 (2·8)	0·71 (0·67-0·75, p<0·001)	0·77 (0·73-0·81, p<0·001)	-
SIMD	5 - least deprived	82594 (96·6)	2891 (3·4)	-	-	-
	4	60493 (96·4)	2234 (3·6)	1·06 (1·00-1·12, p=0·062)	1·05 (0·99-1·11, p=0·106)	1·05 (0·99-1·11, p=0·089)
	3	58327 (96·3)	2256 (3·7)	1·11 (1·04-1·17, p<0·001)	1·07 (1·01-1·13, p=0·016)	1·08 (1·02-1·14, p=0·011)
	2	71941 (96·0)	2984 (4·0)	1·19 (1·12-1·25, p<0·001)	1·10 (1·04-1·16, p<0·001)	1·11 (1·05-1·17, p<0·001)
	1 - most deprived	45065 (95·9)	1940 (4·1)	1·23 (1·16-1·30, p<0·001)	1·12 (1·06-1·19, p<0·001)	1·13 (1·07-1·20, p<0·001)
	SIMD Unknown	6906 (96·7)	234 (3·3)	0·97 (0·84-1·11, p=0·638)	0·99 (0·86-1·13, p=0·880)	-
ED attendances	0 in previous year	275706 (96·9)	8940 (3·1)	-	-	-
	1 in previous year	36861 (94·6)	2117 (5·4)	1·77 (1·69-1·86, p<0·001)	1·66 (1·58-1·74, p<0·001)	1·69 (1·61-1·77, p<0·001)
	2 or more in previous year	12759 (89·6)	1482 (10·4)	3·58 (3·38-3·79, p<0·001)	2·98 (2·80-3·17, p<0·001)	3·05 (2·87-3·25, p<0·001)
Triage category	Medical expected	3321 (97·6)	80 (2·4)	-	-	-
	See and treat	65104 (97·8)	1437 (2·2)	0·92 (0·73-1·16, p=0·452)	1·08 (0·87-1·37, p=0·496)	1·11 (0·88-1·39, p=0·380)
	Standard or Non-urgent	117826 (96·4)	4387 (3·6)	1·55 (1·24-1·95, p<0·001)	1·74 (1·40-2·20, p<0·001)	1·77 (1·42-2·22, p<0·001)
	Urgent	106839 (95·4)	5149 (4·6)	2·00 (1·61-2·52, p<0·001)	2·06 (1·66-2·60, p<0·001)	2·11 (1·69-2·65, p<0·001)
	Very urgent or immediate	19923 (95·8)	875 (4·2)	1·82 (1·46-2·32, p<0·001)	1·76 (1·40-2·24, p<0·001)	1·81 (1·43-2·29, p<0·001)
	Other/Unknown	12313 (95·3)	611 (4·7)	2·06 (1·64-2·63, p<0·001)	2·30 (1·82-2·93, p<0·001)	-
Time of day	0600-1159	86015 (96·6)	2987 (3·4)	-	-	-
	1200-1759	118203 (96·4)	4391 (3·6)	1·07 (1·02-1·12, p=0·005)	1·08 (1·03-1·13, p=0·002)	1·08 (1·03-1·13, p=0·002)
	1800-2359	88153 (96·0)	3718 (4·0)	1·21 (1·16-1·28, p<0·001)	1·23 (1·17-1·29, p<0·001)	1·23 (1·17-1·29, p<0·001)
	0000-0559	32951 (95·8)	1443 (4·2)	1·26 (1·18-1·34, p<0·001)	1·20 (1·12-1·28, p<0·001)	1·19 (1·11-1·27, p<0·001)
Month	January - March	77231 (96·3)	2957 (3·7)	-	-	-
	April - June	81780 (96·2)	3210 (3·8)	1·03 (0·97-1·08, p=0·339)	1·03 (0·98-1·08, p=0·302)	1·03 (0·98-1·08, p=0·292)
	July - September	84104 (96·3)	3258 (3·7)	1·01 (0·96-1·06, p=0·652)	1·02 (0·97-1·08, p=0·407)	1·02 (0·97-1·08, p=0·406)
	October - December	82211 (96·4)	3114 (3·6)	0·99 (0·94-1·04, p=0·681)	0·99 (0·94-1·04, p=0·706)	0·99 (0·94-1·04, p=0·568)
Year	2012	37710 (96·3)	1456 (3·7)	-	-	-
	2013	37949 (96·5)	1387 (3·5)	0·95 (0·88-1·02, p=0·151)	0·91 (0·84-0·98, p=0·014)	0·90 (0·84-0·97, p=0·007)
	2014	39080 (96·3)	1510 (3·7)	1·00 (0·93-1·08, p=0·984)	0·95 (0·88-1·02, p=0·154)	0·93 (0·87-1·00, p=0·062)
	2015	38224 (96·5)	1403 (3·5)	0·95 (0·88-1·02, p=0·184)	0·90 (0·83-0·97, p=0·004)	0·88 (0·81-0·95, p=0·001)
	2016	40819 (96·3)	1588 (3·7)	1·01 (0·94-1·08, p=0·838)	0·94 (0·87-1·01, p=0·098)	0·92 (0·85-0·99, p=0·018)
	2017	41144 (96·1)	1656 (3·9)	1·04 (0·97-1·12, p=0·257)	0·98 (0·91-1·05, p=0·579)	0·95 (0·88-1·02, p=0·150)
	2018	43417 (96·2)	1726 (3·8)	1·03 (0·96-1·11, p=0·421)	1·00 (0·93-1·08, p=0·964)	0·97 (0·90-1·04, p=0·333)
	2019	46983 (96·3)	1813 (3·7)	1·00 (0·93-1·07, p=0·987)	0·99 (0·92-1·07, p=0·847)	0·95 (0·88-1·02, p=0·141)

Logistic regression models (univariable and multivariable) using 7-day reattendance as the outcome, for patients discharged alive from ED. Number in dataframe = 337865, Number in model = 337851, Missing = 14, AIC = 104294·2, C-statistic = 0·632, H&L = Chi-sq(8) 18·81 (p=0·016). In multiple imputation model there were 337865 patients included· Missing values for Sex (n=10 (0%)), SIMD (n=7140 (2·1%)), Ethnicity (n=55073 (16·3%)), Triage category (n=12924 (3·8%)), and presentation time (n=4 (0%)) have been imputed using a pool of 10 iterations.

eTable 8: Interaction terms between age and multimorbidity for primary and secondary outcomes. Table summarizing significance testing in regression models using interaction terms for age category (<65 years vs ≥65 years) with multimorbidity for 4 different outcomes: 30-day mortality, 7-day reattendance and hospital admission (multivariable logistic regression models), and ED length of stay (multivariable linear regression models).

**Outcome**	**Interaction term**	**p-value**
30-day Mortality	Multimorbidity with Age	**p<0·001**
7-day Reattendance	Multimorbidity with Age	**p<0·001**
Hospital Admission	Multimorbidity with Age	**p<0·001**
ED Length of stay (hours)	Multimorbidity with Age	**p=0·001**

eFigure 1: Count of long-term conditions (LTCs) by age, sex and SIMD. Proportions of patients with different number of Elixhauser conditions (derived using diagnosis codes from SMR01 and SMR06 over a look-back of 5 years prior to ED attendance) as a proportion of the cohort, stratified by age category (groups of 10 years from 20-90+), sex and SIMD quintile. LTC=Long-term condition; SIMD=Scottish Index of Multiple Deprivation.

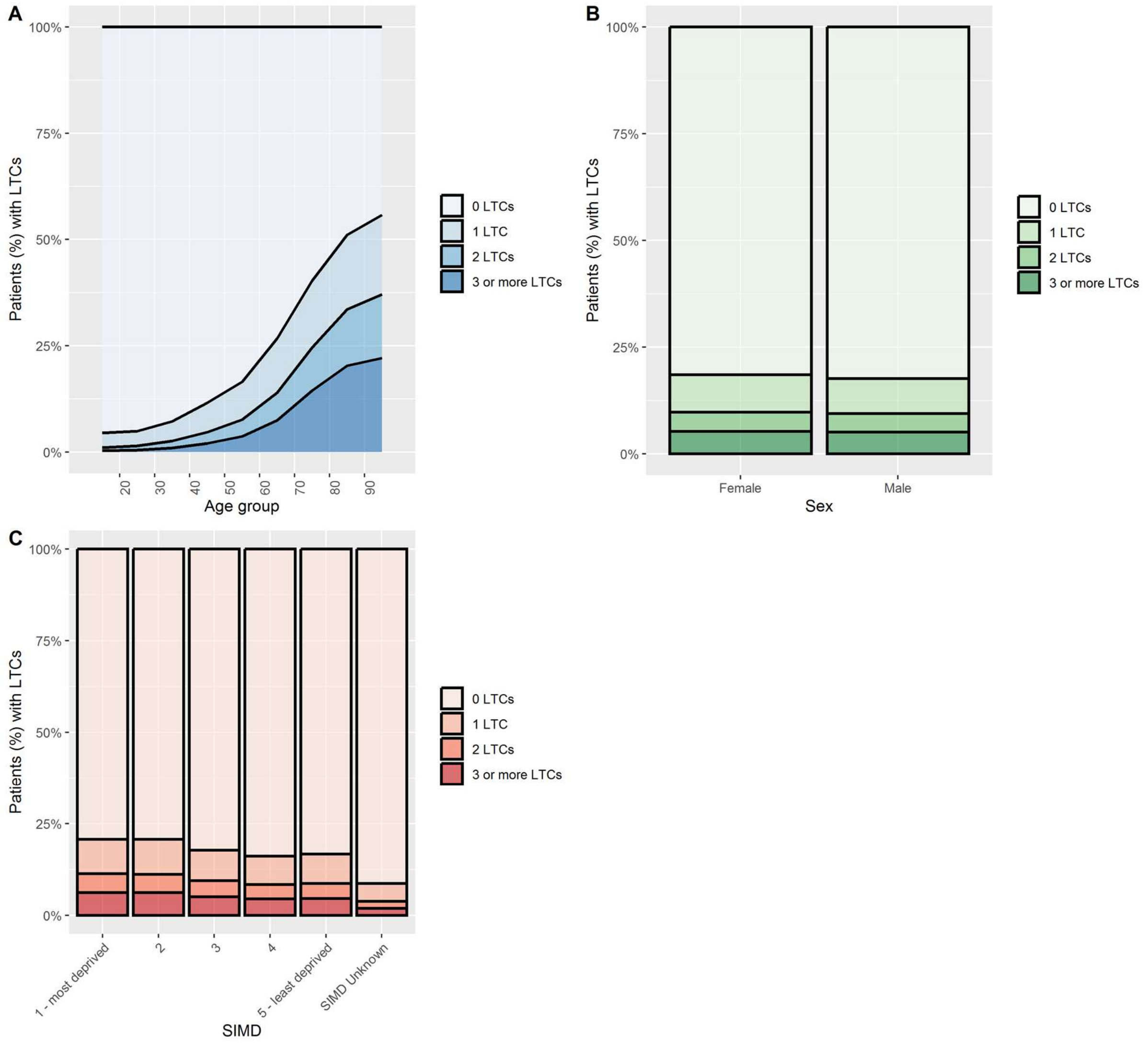

eFigure 2: Cumulative incidence of ED reattendance, stratified by multimorbidity and age. Panel A is stratified by multimorbidity. Panel B is stratified by multimorbidity and age category (<65 years old vs ≥65 years old).

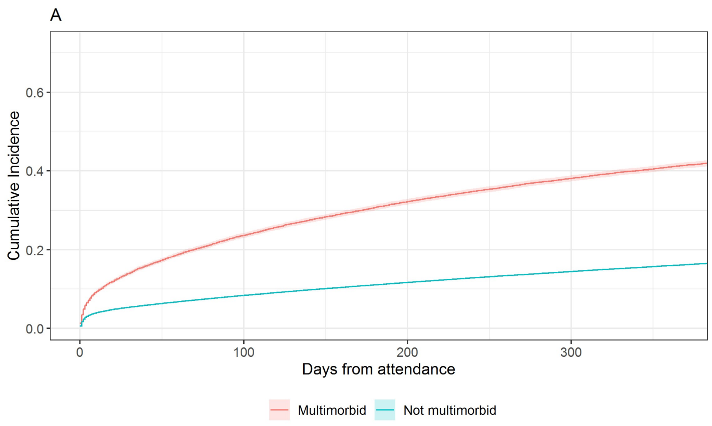

Multimorbid					
At Risk	16874	11940	9978	8517
Events	223	3943	5306	6210
Not multimorbid				
At Risk	320991	281811	259837	240718
Events	1743	26510	36367	44347

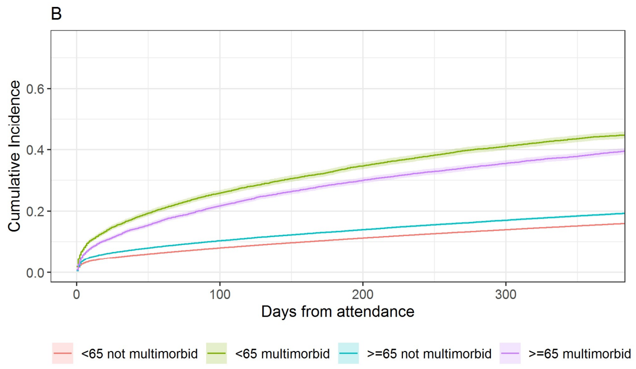

At Risk				
<65 not multimorbid	278482	245965	227384	211259
<65 multimorbid	7861	5515	4659	4029
>=65 not multimorbid	42509	35846	32453	29459
>=65 multimorbid	9013	6425	5319	4488
Events				
<65 not multimorbid	1504	22143	30584	37416
<65 multimorbid	144	2010	2668	3124
>=65 not multimorbid	239	4367	5783	6931
>=65 multimorbid	79	1933	2638	3086

eFigure 3: Associations between individual conditions and mortality. Panel of Forest plots showing the multivariable associations between individual Elixhauser conditions (derived using diagnosis codes from SMR01, SMR04 and SMR06 over a look-back of 5 years prior - hospital attendance) and 30-day mortality using logistic regression, controlling for age, sex, ethnicity, SIMD, triage category, previous ED attendances, time of day, month and year. Conditions are arrange by ascending OR point estimate. *PUD=Peptic Ulcer Disease (without GI bleeding)· PVD=Peripheral Vascular Disease, AIDS/HIV=Acquired Immunodeficiency Syndrome/Human Immunodeficiency Virus·

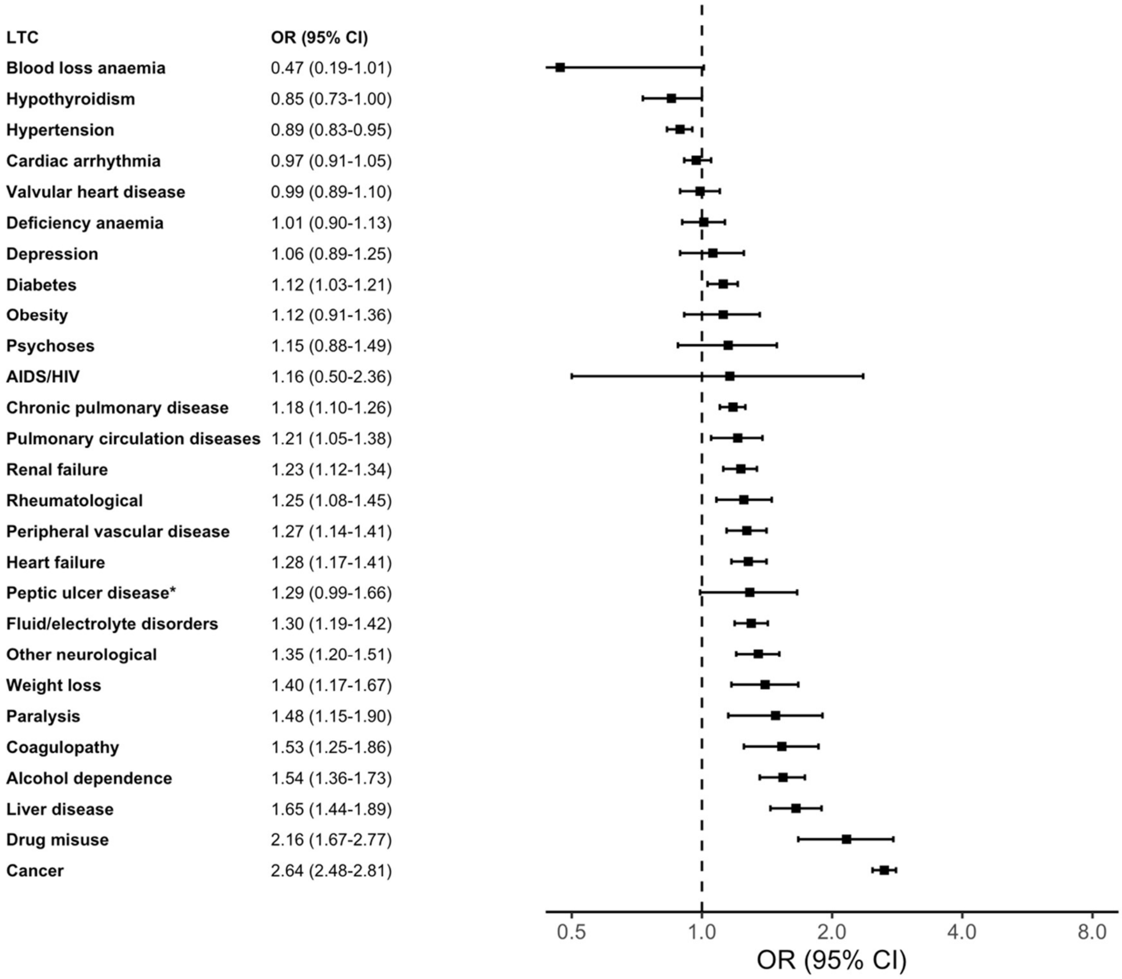

eFigure 4: Associations between individual conditions and mortality, stratified by age category: A shows people <65 years old; B shows people ≥65 years old. Panel of Forest plots showing the multivariable associations between individual Elixhauser conditions (derived using diagnosis codes from SMR01, SMR04 and SMR06 over a look-back of 5 years prior - hospital attendance) and 30-day mortality using logistic regression, controlling for age, sex, ethnicity, SIMD, triage category, previous ED attendances, time of day, month and year. Conditions are arranged by ascending OR point estimate in the whole population (eFigure 3) for comparison. *PUD=Peptic Ulcer Disease (without GI bleeding)· PVD=Peripheral Vascular Disease, AIDS/HIV=Acquired Immunodeficiency Syndrome/Human Immunodeficiency Virus·

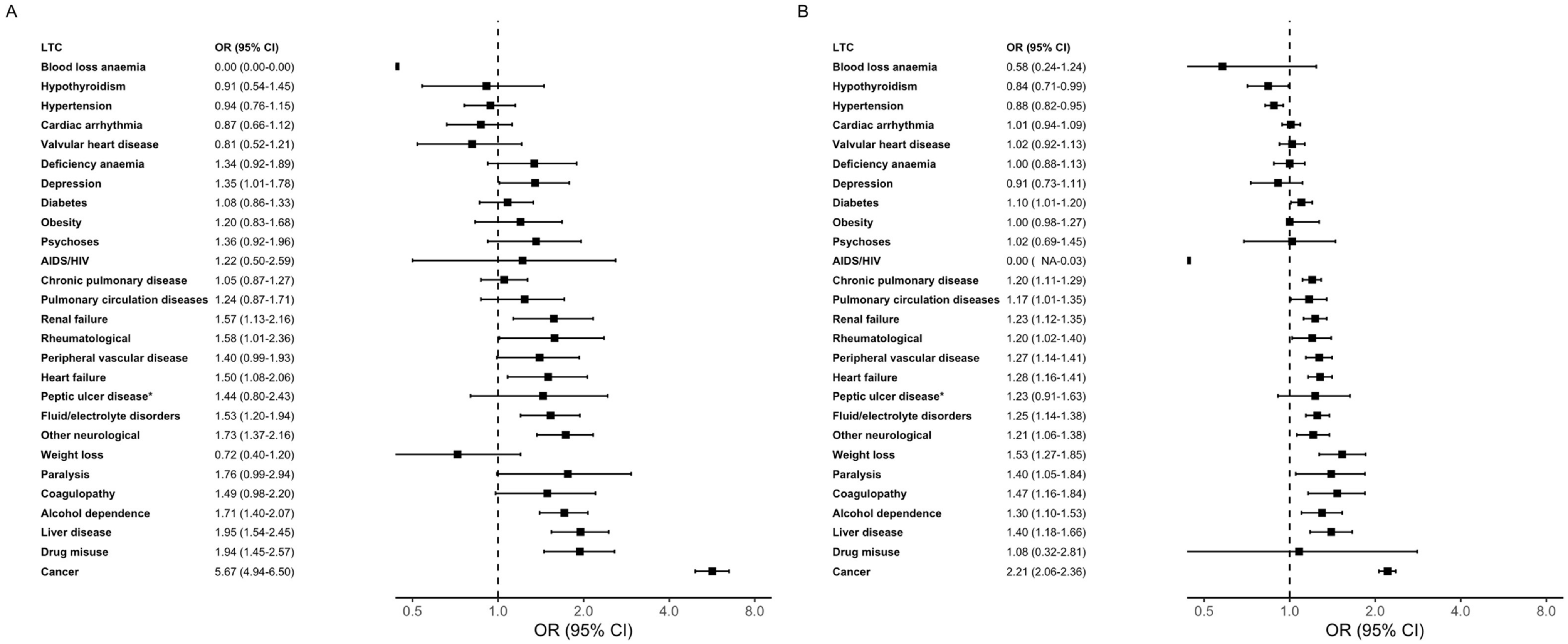

eFigure 5: Age as an effect modifier of the association between multimorbidity and mortality. Age is modelled as a continuous term in the interaction term in the multivariable model.

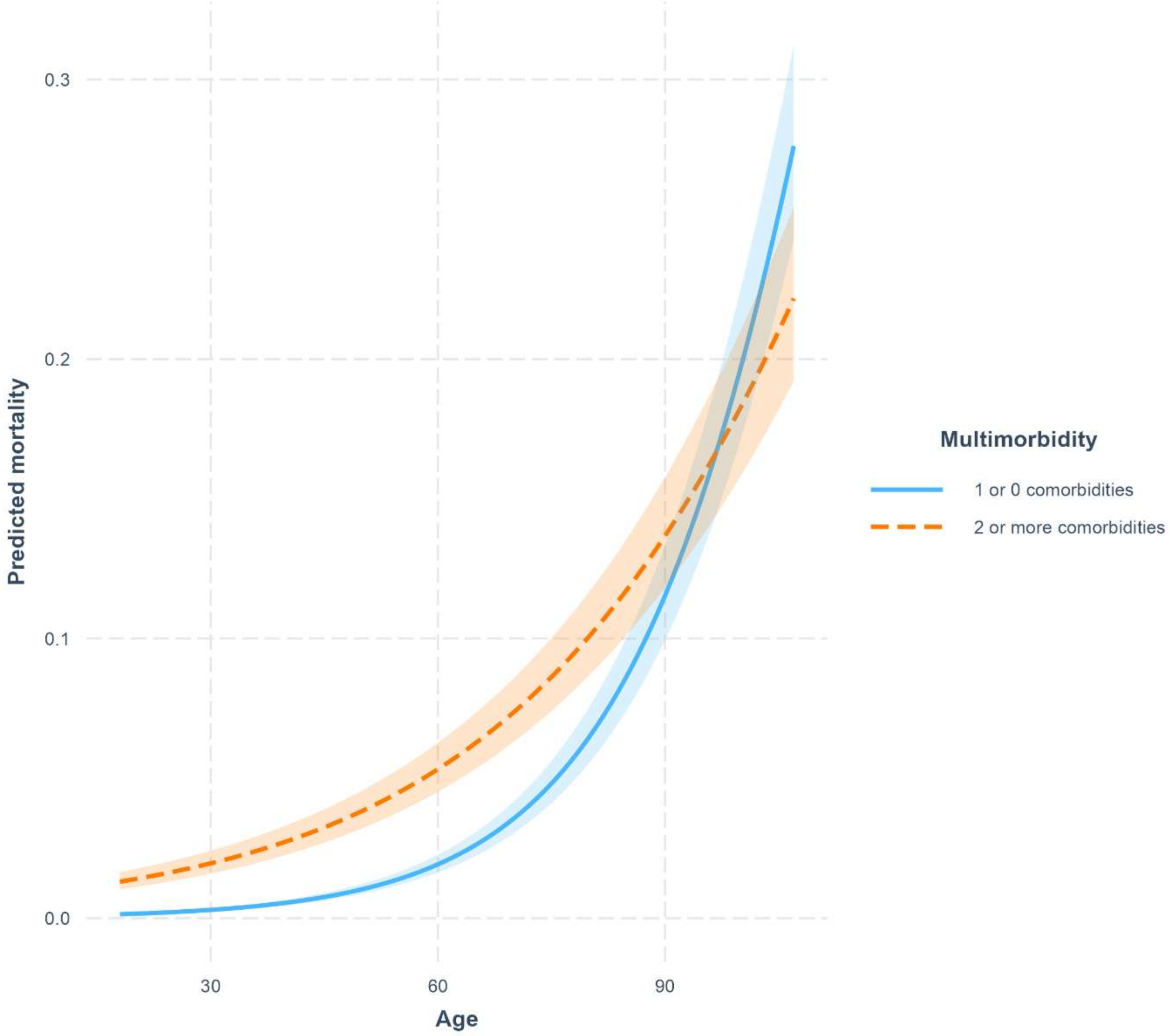

Supplement: online supplemental file 2 [file bmjmed-3-1-s002.pdf]
