# Supplementary material for: Multimorbidity and adverse outcomes following emergency department attendance: population based cohort study
Source: BMJ Med. 2024 Aug 16;3(1):e000731. doi: 10.1136/bmjmed-2023-000731 (PMC11344864; doi:10.1136/bmjmed-2023-000731)
Supplement: Supplementary file 3 [file bmjmed-2023-000731.reviewer_comments.pdf]

PEER REVIEW HISTORY

BMJ Medicine publishes all reviews undertaken for accepted manuscripts. Reviewers are asked to complete a checklist review form and are provided with free text boxes to elaborate on their assessment. These free text comments are reproduced below.

ARTICLE DETAILS

**TITLE (PROVISIONAL)**	Multimorbidity and adverse outcomes following Emergency Department attendance: a cohort study
**AUTHORS**	Blayney, Michael; Reed, Matthew; Masterson, John; Anand, Atul; Bouamrane, Matt; Fleuriot, Jacques; Luz, Saturnino; Lyall, Marcus; Mercer, Stewart; Mills, Nicholas; Shenkin, Susan; Walsh, Timothy; Wild, Sarah H; Wu, Honghan; McLachlan, Stela; Guthrie, Bruce; Lone, Nazir I

VERSION 1 - REVIEW

**REVIEWER 1**	Yordanov, Youri; Sorbonne Universite, Emergency Department.<br>Competing Interest: None
**REVIEW RETURNED**	07-Oct-2023

**GENERAL COMMENTS**	The paper titled "Multimorbidity in Emergency Department Attendees: Prevalence, Associations, and Outcomes" provides an interseting analysis of the prevalence of multimorbidity in ED attendees, along with its associations with mortality, length of stay or other interesting variables for EM specialists.<br><br>The study is nicely written, well-structured, and the methodology is sound. The used databases adds credibility to the findings. However, there are several points that would need calrification<br><br>Definition Multimorbidity: this paper employs the Elixhauser list of conditions to define multimorbidity, with modifications. The rationale behind these modifications should be discussed as these modification might have a major impact on the results of the analyses.<br><br>Databases: did the author had a quality assurance measure to ensure the accuracy and reliability of the data?<br><br>The statistical methods used are appropriate and appear robust, including the missing data management.<br><br>Age stratification : 65 years is a more and more debated limit. Did the authors considered using the 75 years old limit? And how would that age limit affect the results?<br><br>Last comment: the paper could benefit from a paragraph or a couple of lines on how this study can have practical implications in clinical practice, bu shedding a specific light on a fragile population of patients attending EDs.

**REVIEWER 2**	Chocano-Bedoya, Patricia. Competing Interest: None
**REVIEW RETURNED**	27-Nov-2023

**GENERAL COMMENTS**	The risk of adverse outcomes in people with multimorbidity is an important topic. While the paper is well written and clarifies the need for this research well, I have some questions regarding the statistical methods, which focused on logistic regression, whereas with this type of data other methods could be considered:<br><br>Primary outcome: Mortality within the first 30 days. I think the authors should have considered using survival analysis to evaluate this outcome.<br><br>Secondary outcomes and other results:<br>In the first line of the results part of the abstract the authors mention "451,291 people had 1,273,937 attendances to EDs during the study period". It would be far more interesting to report a rate here. Rates can be calculated with Poisson regression, or Negative Binomial regression (depending if the assumptions can be fulfilled). This type of analysis would allow the authors to evaluate for example the characteristics of patients that had higher rates of ED admissions, or among those with multimorbidity, the characteristics that led to an increase of negative outcomes.<br>Discharge destination on the other hand, could be better evaluated using multinomial logistic regression, as there are several options where a patient can be discharged.<br><br>Minor revisions: All acronyms should be described at first use, including the abstract. There are so many acronyms throughout the paper that it is a bit hard to follow.<br>For the statistical analysis, it is best to cite the software and packages at the end of the section.

**REVIEWER 3**	Riley, Richard; University of Birmingham, Institute of Applied Health Research. Competing Interest: None
**REVIEW RETURNED**	20-Dec-2023

**GENERAL COMMENTS**	Thank you for the opportunity to review this article. It is clear that this article is well written and the authors have worked hard to produce it for our review. I have examined from a statistical perspective and have some comments, as follows:<br><br>1) In the context of a well written paper, I did not quite understand what the paper added. Is it not known that multimorbidity is associated with worse outcomes? Surely it is – so why should we be surprised that those with multimorbidity have worse outcomes? I see the novelty sold here is the ED setting – but really the question should be whether those with multimorbidity do worse than expected, not that they do worse than those without multimorbidity. This is a subtle point but I don't see this addressed.<br>2) The authors say they adjusted for confounding, but I expected there to be more of a causal pathway process here to selection of variables, rather than simply selected variables and adjusting for them. I think this also raises the issue about whether this study is aiming to establish causality, or whether it is about whether multimoribidy is prognostic. It is easier to demonstrate the latter: essentially here multimorbidity is prognostic after adjusting for other prognostic factors here. But establishing causality – well I think this needs more causal inference methodology. So I think the study is best reframed as a prognostic factor study, if the causal inference framework cannot be established.<br>3) How do we know that the set of adjustment factors is sensible or enough? Those with multimorbidity will also have many other characteristics that are prognostic of poor outcomes – how can we

	be sure that these are all covered by the adjustment factors included? In the framing of a prognostic question, how do we know that all existing prognostic factors are accounted for?<br>4) The subgroup approach is not appropriate. There is no reason for dichotomising age at 65 years. If age is truly of interest, in regards to the impact on the prognostic effect of multimorbidity, then it should be modelled as a continuous variable, allowing for potential non-linear relationships with splines or polynomials.<br>5) There are two ED departments included, but I can't see this clustering accounted for in the modelling<br>6) Those with multimorbidity are more likely to be coming to the ED more than once, I suspect. But the authors randomly only select one occurrence. Is this not diluting the fact that those with multimorbidity have a more complex pathway? Again is this a source of confounding that is not accounted for?<br>7) A related point is that some individuals will attend twice, firstly without multimorbidity, and secondly (at a later date) with multimorbidity. Is this not an opportunity to remove some aspects of confounding by accounting for this patient level clustering with multiple responses for each individual?<br><br>In summary, confusion needs to be resolved about whether this a causal inference or prognostic factor study, and the modelling of age needs to be improved, for this to be considered further at BMJ Medicine. Regardless, I hope these comments are constructive for the authors moving forward.

VERSION 1 – AUTHOR RESPONSE

	**Reviewer/Editor Comments**	**Response**
	**Reviewer: 1**<br>**Dr. Youri Yordanov, Sorbonne Universite**<br>The paper titled "Multimorbidity in Emergency Department Attendees: Prevalence, Associations, and Outcomes" provides an interesting analysis of the prevalence of multimorbidity in ED attendees, along with its associations with mortality, length of stay or other interesting variables for EM specialists. The study is nicely written, well-structured, and the methodology is sound. The used databases adds credibility to the findings. However, there are several points that would need clarification	3
1	Definition Multimorbidity: this paper employs the Elixhauser list of conditions to define multimorbidity, with modifications. The rationale behind these modifications should be discussed as these modification might have a major impact on the results of the analyses.	The only modifications made to Elixhauser comorbidities was to merge two distinct categories relating to a single long-term condition into one category e.g. "Hypertension without complications" and "Hypertension with complications" were merged into a single category "hypertension". Three long term conditions were modified in this way: cancer (with/without metastases), hypertension (with/without complications),

		diabetes (with/without complications). However, these two -level long-term conditions are, by definition, mutually exclusive i.e. it is not possible to have both hypertension with complications and without complications at the time of ED attendance.<br>Therefore, as multimorbidity is defined by the count of long term conditions, this modification had no impact on the frequency of multimorbidity in the population, or the associations with outcomes.
2	Databases: did the author had a quality assurance measure to ensure the accuracy and reliability of the data?	We have added information about accuracy and reliability of the data sources in the methods section and added appropriate references (lines 16-19, p7/66).
3	The statistical methods used are appropriate and appear robust, including the missing data management.<br>Age stratification : 65 years is a more and more debated limit. Did the authors considered using the 75 years old limit? And how would that age limit affect the results?	We planned an age stratification threshold of 65 years *a priori*, following consultation with clinicians and patient/public partners. This threshold has particular significance in the UK, as access to health services and social care services are often determined on age-based criteria. Older patients with multimorbidity, due to the overlap of multimorbidity with frailty, are more likely to be assessed by generalist services familiar with managing acute conditions in the context of multimorbidity, such as Medicine of the Elderly (Geriatrics) services and Acute Frailty services, as this age threshold is one of eligibility criteria used when screening acute admissions for these services.[1] These services have a strong multidisciplinary ethos and good connections with community services and social care. Social
		care services may also be required for those with care needs in order to facilitate discharge from the emergency department. However, there is a growing gap between care need and care provision in younger populations.[2] For this reason, we deemed it important to present a clear description of multimorbidity in these age-defined subgroups, and its association with processes within ED and outcomes. We have amended the manuscript to make the rationale for this age threshold clearer. (lines 17-20, p6/66; lines 52-56 p8/66; lines 3-7 p9/66).<br><br>1. Knight, T., Atkin, C., Martin, F.C. et al. Frailty assessment and

		acute frailty service provision in the UK: results of a national 'day of care' survey. BMC Geriatr 22, 19 (2022). Doi:10.1186/s12877-021- 02679-9<br>2. Idriss O, Allen L, Alderwick H. Social care for adults aged 18–64. Health Foundation; 2020. Doi:10.37829/HF-2020-P02
4	Last comment: the paper could benefit from a paragraph or a couple of lines on how this study can have practical implications in clinical practice, but shedding a specific light on a fragile population of patients attending EDs.	We thank the Reviewer for raising this. We have amended the Discussion and highlighted the paragraphs which make clearer the practical implications for clinical practice and policy. (lines 6-26, p16/66).
	**Reviewer: 2**<br>**Dr. Patricia Chocano-Bedoya**<br>The risk of adverse outcomes in people with multimorbidity is an important topic. While the paper is well written and clarifies the need for this research well, I have some questions regarding the statistical methods, which focused on logistic regression, whereas with this type of data other methods could be considered:	
5	Primary outcome: Mortality within the first 30 days. I think the authors should have considered using survival analysis to evaluate this outcome.	In the context of a fixed-timepoint outcome, survival methods are of greatest benefit when incomplete follow-up is present in a dataset, or when there is necessity to maximise statistical power (for example, with small sample sizes).[1] Our dataset contained complete follow-up for all 451,291 patients at 30 days, and the event frequency for the primary outcome was 1.9% (n=8516). Furthermore, as the primary outcome was relatively uncommon, the odds ratio reported for the primary analysis approximates to the risk ratio, aiding clinical interpretation. For these reasons, we
		suggest that the benefits of survival methods over our chosen approach are likely to be minimal. However, we would be happy to revise analyses if advised by the editorial team.<br><br>1. Annesi, I., Moreau, T., & Lellouch, J. (1989). Efficiency of the logistic regression and Cox proportional hazards models in

		longitudinal studies. Statistics in medicine, 8(12), 1515-1521.
6	Secondary outcomes and other results:<br>In the first line of the results part of the abstract the authors mention "451,291 people had 1,273,937 attendances to EDs during the study period". It would be far more interesting to report a rate here. Rates can be calculated with Poisson regression, or Negative Binomial regression (depending if the assumptions can be fulfilled). This type of analysis would allow the authors to evaluate for example the characteristics of patients that had higher rates of ED admissions, or among those with multimorbidity, the characteristics that led to an increase of negative outcomes.	We agree that reporting rates may be of interest for determining which patients in the population had higher rates of ED attendances (if we have correctly understood the Reviewer's comment). To calculate these rates, we would also need to know the denominator population at risk of ED attendance, which we unfortunately did not have access to. Furthermore, attendances to ED in hospitals in the Lothian region are not limited by geographical boundaries, further complicating calculating this rate. In terms of adverse outcomes, our chosen outcomes were binary and continuous variables, allowing us to use logistic and linear regression modelling approaches rather than count models.
7	Discharge destination on the other hand, could be better evaluated using multinomial logistic regression, as there are several options where a patient can be discharged.	Thank you for your suggestion of modelling discharge destination using an alternative approach. Whilst there are several options available for discharge destination for patients, our collaborators indicated that the most important outcome within these categories was likelihood of hospital admission (vs not), which was determined from our wider engagement with clinicians (including colleagues from ED, primary care, and inpatient specialties) and patients; this was the rationale for modelling a binary outcome rather than using a multinomial model. We have therefore retained our current analytical approach to maximise relevance to the intended audiences of the article.
8	Minor revisions: All acronyms should be described at first use, including the abstract. There are so many acronyms throughout the paper that it is a bit hard to follow.	Many thanks for highlighting this. We have amended the manuscript to describe acronyms at first use, and attempted to minimise their use.
9	For the statistical analysis, it is best to cite the software and packages at the end of the section.	We have cited the software packages used at the end of the statistical analysis section as suggested. (lines 29-30, p9/66).
	**Reviewer: 3**<br>**Prof. Richard Riley, University of Birmingham**	

	Thank you for the opportunity to review this article. It is clear that this article is well written and the authors have worked hard to produce it for our review. I have examined from a statistical perspective and have some comments, as follows:	
10	1) In the context of a well written paper, I did not quite understand what the paper added. Is it not known that multimorbidity is associated with worse outcomes? Surely it is – so why should we be surprised that those with multimorbidity have worse outcomes? I see the novelty sold here is the ED setting – but really the question should be whether those with multimorbidity do worse than expected, not that they do worse than those without multimorbidity. This is a subtle point but I don't see this addressed.	Whilst previous epidemiological studies investigating multimorbidity have reported the association between multimorbidity and worse outcomes, the novelty of the study is not solely framed around replicating this association in the ED context. There is little recognition of multimorbidity in the ED setting, and almost no descriptive epidemiology of this population. The importance of descriptive epidemiology and its epistemic contribution in such a context is increasingly recognised.[1,2]<br><br>The ED context is distinct from other health care and community contexts, with over-representation of those from deprived backgrounds for whom ED may be their only point of contact with health services, and, most importantly, the need for time-critical decision-making and treatment. This context is the primary novelty of the study, but the study also adds to literature in the area through extending outcomes beyond mortality to process measures to better understand how multimorbidity impacts on these measures.<br><br>It is this detailed description, using data not easily obtainable from most datasets, which we believe makes this a valuable addition to current knowledge in the field.<br><br>We have amended the Key Messages and Discussion section to make this clearer. (lines 54-56, p4/66; lines 3-6, p5/66; lines 24-26, p15/66; lines 6-26 p17/66).<br><br>1. Fox MP, Murray EJ, Lesko CR, Sealy-Jefferson S. On the Need to Revitalize Descriptive Epidemiology. Am J Epidemiol. 2022 Jun 27;191(7):1174-1179. doi: 10.1093/aje/kwac056.<br>2. Platt RW. Invited Commentary: The Importance of Descriptive Epidemiology. *Am J Epidemiol*. 2022;191(12):2071-2072.

		doi:10.1093/aje/kwac153
11	2) The authors say they adjusted for confounding, but I expected there to be more of a causal pathway process here to selection of variables, rather than simply selected variables and adjusting for them. I think this also raises the issue about whether this	Thank you for drawing attention to this ambiguity in our study framing. We have now made clear that we were not intending to draw causal inference from our study - which is often challenging when a population is selected through contact with the health service due to selection bias caused by conditioning on a collider - but highlighting the prognostic effect of multimorbidity on a range of outcomes. We have amended any language that implies that causal inference was our aim, and

	study is aiming to establish causality, or whether it is about whether multimorbidity is prognostic. It is easier to demonstrate the latter: essentially here multimorbidity is prognostic after adjusting for other prognostic factors here. But establishing causality – well I think this needs more causal inference methodology. So I think the study is best reframed as a prognostic factor study, if the causal inference framework cannot be established.	added a sentence to the methods section to state explicitly that we were not aiming to draw causal inference from the reported associations.<br>(lines 43-46, p8/66).
12	3) How do we know that the set of adjustment factors is sensible or enough? Those with multimorbidity will also have many other characteristics that are prognostic of poor outcomes – how can we be sure that these are all covered by the adjustment factors included? In the framing of a prognostic question, how do we know that all existing prognostic factors are accounted for?	We agree that this question should be posed of all observational research with aetiological or predictive components, regardless of whether framed using causal inference or a prognostic approach. There is a well-recognised trade-off between access to whole population-level datasets (thus minimising selection bias) and sufficient ascertainment of relevant covariates (increasing confounding bias in the context of casual inference). In our previous work, we had the benefit of additional funding to undertake qualitative research to triangulate qualitative and quantitative findings, which allowed us to identify important factors not included in the prediction model.[1] In the absence of qualitative research, we were reliant on our knowledge of the wider literature and engagement with clinicians and PPI groups to identify a "sensible" set of prognostic factors. We highlighted one factor which was not adequately captured (severity of illness).<br><br>We have expanded the limitations section to include other factors which we were unable to include, likely to be associated with outcomes (and multimorbidity status).<br>(lines 17-23, p16/66).<br><br>1. Donaghy E, Salisbury L, Lone NI, et al. Unplanned early hospital readmission among critical care survivors: a mixed methods study of patients and carers. BMJ Qual Saf. 2018 Nov;27(11):915-927. doi: 10.1136/bmjqs-2017-007513.
13	4) The subgroup approach is not appropriate. There is no reason for dichotomising age at 65 years. If age is truly of interest, in regards to the impact on the prognostic effect of multimorbidity, then it should be modelled as a continuous variable, allowing for	We agree that, from a statistical perspective, most information would be retained if age was modelled as a continuous term in effect modification analyses that explored whether the prognostic effect of multimorbidity varied across ages. Indeed, cognisant of this general principle of avoiding dichotomisation of continuous variables, we ensured

		age was represented in models as a continuous *prognostic* covariate in models (having assessed linearity).
	potential non-linear relationships with splines or polynomials.	However, the dichotomised age cut off in effect modification analyses is of most relevance to clinical practice and service configurations when considering the prognostic effect of multimorbidity within subgroups. We have outlined the reasons for this below, which are grounded in the reality of health and care service organisation and the experience of clinicians and patients with complex conditions.<br><br>A key aim of our study was, in addition to describing characteristics, processes and outcomes in relation to multimorbidity for the whole population, to specifically report and contrast these findings for older and younger adults. Patients under 65 often do not have access to the same multidisciplinary services that those aged 65 and older have, which may be particularly problematic for people under 65 attending with complex needs. This means that there is a real-world distinction between a multimorbid 64 year old and 65 year old when it comes to their interaction with health and care services in the acute care context. In contrast, this distinction does not exist for someone aged 42 compared with someone aged 43. It is this age-group contrast, rather than how the multimorbidity-outcome association varies over the entire range of age, that our collaborators indicated was of most importance, and would translate into clinical, service and policy implications.<br><br>We acknowledge that we have not made the rationale for this approach sufficiently clear, and have amended the Introduction and Methods section to make this explicit. For the reasons we have set out, we currently have not included analyses which model the age*multimorbidity interaction with age as a continuous term, would be happy to provide these in the supplementary materials in addition to the current analyses if of interest. (lines 17-20, p6/66; lines 52-56 p8/66; lines 3-7 p9/66).

14	5) There are two ED departments included, but I can't see this clustering accounted for in the modelling	We unfortunately did not have a marker of ED department in the dataset. We have amended the discussion to highlight this limitation and its potential consequences. (lines 17-23, p16/66).
15	6) Those with multimorbidity are more likely to be coming to the ED more than once, I suspect. But the authors randomly only select one occurrence. Is this not diluting the fact that those with multimorbidity have a more complex pathway? Again is this a source of confounding that is not accounted for?	We opted to sample a random ED attendance rather than the first attendance. The main reason for doing this was that, over the relatively long study period, first attendees in the final year would be relatively “abnormal” individuals who had not attended an ED department for the preceding 8 years (a phenomenon which mirrors the mechanism underpinning the "prevalent pool effect" previously described). We did have access to an aggregated variable of number of ED attendances in the previous year and number of hospital admissions in the previous year, which were included in models. Given that, as the Reviewer suggests, patients with multimorbidity are more likely to attend

		more frequently, the inclusion of these covariates allows us to conclude that the presence of multimorbidity has an additional prognostic role over and above previous health care use.
16	7) A related point is that some individuals will attend twice, firstly without multimorbidity, and secondly (at a later date) with multimorbidity. Is this not an opportunity to remove some aspects of confounding by accounting for this patient level clustering with multiple responses for each individual?	Thank you for suggesting this interesting approach. This will be a useful consideration in our future work which moves beyond identifying prognostic effects to better understand causal mechanisms in relation to multimorbidity and outcomes within emergency care contexts.
17	In summary, confusion needs to be resolved about whether this a causal inference or prognostic factor study, and the modelling of age needs to be improved, for this to be considered further at BMJ Medicine. Regardless, I hope these comments are constructive for the authors moving forward.	Thank you for these constructive comments. We hope that our revisions goes some way to resolving these issues by providing: greater clarity relating to the framing of the study as one investigating the prognostic effect of multimorbidity; a clear indication of how these findings add to the existing knowledge; and an explicit justification for the choice of a subgroup approach when exploring effect modification (in contrast to modelling multimorbidity*age interaction as a continuous variable) in the context of the current age-based cut-offs used in clinical service organisation and ease of access to services within health and social care sectors.
	**Editor(s)' Comments to Author (if any):**	
18	:: Concerns were raised by our statistical editor regarding the purpose of the study, namely whether this was intended as a causal inference study or a prognostic study. Please can you clarify your intention.	We have amended text in the Methods section and removed any language which can be interpreted as causal throughout the manuscript to clarify our intention. (lines 43-46, p8/66).
19	:: If intended as a prognostic study, it would be helpful to emphasise more clearly what these findings add to existing knowledge.	We have amended the Key Messages and Discussion to emphasise more clearly how our study adds to the existing literature and implications. (lines 54-56, p4/66; lines 3-6, p5/66; lines 24-26, p15/66; lines 6-26 p17/66).
20	:: The research is described as patient-centred, how did authors ascertain that these were the outcomes that mattered most to patients?	During the design phase of the study, we consulted a PPI group to consider options for possible outcomes in the study. Within the limitations of the data available, the PPI group agreed that these outcomes were important to them. We have amended the methods section to reflect this.

		(lines 34-36, p9/66).
21	:: Multimorbidity in the study cohort was 9.6%, which is lower than other estimates of multimorbidity prevalence. This is acknowledged in the discussion, but it would be useful to understand	We have amended the discussion, highlighting a recently published paper from our group which quantifies the difference in prevalence depending on data source, and its impact on association with outcomes.<br>(lines 24-31, p16/66)
	the possible reasons for this and any implications for generalisability.	
22	:: In the supplementary material, it shows that some conditions seem to be associated with a lower risk of mortality, would it be possible to comment on this?	There is only one condition – hypertension – associated with lower mortality for which the 95% confidence interval does not include unity. This is most likely to be due to two phenomena. First, ascertainment of long-term conditions from hospital records generally represents both the presence of the conditions *and* the fact that the condition has precipitated an acute hospital admission.<br>However, for milder conditions, such as hypertension or hypothyroidism, the condition is unlikely to be the primary cause that precipitated previous hospital admissions, but more likely to be coded in the field capturing "other" conditions. Second, the relationship between each long-term condition and outcomes is conditioned on attending the ED. This selection bias can distort the long-term condition-outcome association depending on the strength of the association between the individual condition and ED attendance and the association between outcome (or ancestors of the outcome) and ED attendance, increasingly referred to as "collider bias".[1] We have not added this explanation to the discussion for reasons of space, but would be happy to do so if you feel this would be helpful for readers.<br><br>1. Russell, C.D., Lone, N.I. & Baillie, J.K. Comorbidities, multimorbidity and COVID-19. Nat Med 29, 334–343 (2023). https://doi.org/10.1038/s41591-022-02156-9

VERSION 2 – REVIEW

**REVIEWER 3**	Riley, Richard; University of Birmingham, Institute of Applied Health Research. Competing Interest: None
**REVIEW RETURNED**	22-Apr-2024

**GENERAL COMMENTS**	I thank the authors for a very detailed response and for revising the article so well, including framing it as a prognostic factor study, and for explaining more about the rationale for the study and various modelling choices. It is much improved. Two minor comments remain:<br><br>1) Please do add the age as a continuous variable analysis to the paper at least in supplementary materials.<br>2) In regards to the focus being on prognosis and not causation, I would not use the term ‘exposure’ to define multimorbidity

VERSION 2 – AUTHOR RESPONSE

Many thanks for these further comments.
We have added a figure to the supplement (eFigure 5) to illustrate the age-multimorbidity interaction for the primary outcome with age modelled as a continuous variable, as suggested by Prof Riley.
We have also replaced the term "exposure" with "covariate".
In addition to these changes, we have amended the manuscript to adhere the formatting amendments (1-5).
